# Expression of *FLOWERING LOCUS C* and a frameshift mutation of this gene on chromosome 20 differentiate a summer and winter annual biotype of *Camelina sativa*

**DOI:** 10.1002/pld3.60

**Published:** 2018-07-09

**Authors:** James V. Anderson, David P. Horvath, Münevver Doğramaci, Kevin M. Dorn, Wun S. Chao, Erin E. Watkin, Alvaro G. Hernandez, M. David Marks, Russ Gesch

**Affiliations:** ^1^ Sunflower and Plant Biology Research Unit USDA‐ARS, Red River Valley Agricultural Research Center Fargo North Dakota; ^2^ Sanford School of Medicine Internal Medicine Department University of South Dakota Sioux Falls South Dakota; ^3^ Department of Plant Pathology Kansas State University Manhattan Kansas; ^4^ Department of Crop Sciences 2608 Institute for Genomic Biology, and Roy J. Carver Biotechnology Center University of Illinois Urbana Illinois; ^5^ Department of Plant Biology University of Minnesota St. Paul Minnesota; ^6^ USDA‐ARS, North Central Soil Conservation Research Laboratory Morris Minnesota

**Keywords:** camelina, flowering time, genomics, vernalization

## Abstract

The nature of the vegetative to reproductive transition in the shoot apical meristem of *Camelina sativa* summer annual cultivar CO46 and winter annual cultivar Joelle was confirmed by treating seedlings with or without 8 weeks of vernalization. True to their life cycle classification, Joelle required a vernalization treatment to induce bolting and flowering, whereas CO46 did not. In this study, whole genome sequence, RNAseq, and resequencing of PCR‐amplified transcripts for a key floral repressor were used to better understand factors involved in the flowering habit of summer and winter biotypes at the molecular level. Analysis of transcriptome data indicated that abundance for one of the three genes encoding the floral repressor *FLOWERING LOCUS C* (*FLC*; Csa20 g015400) was 16‐fold greater in Joelle compared to CO46 prior to vernalization. Abundance of this transcript decreased only slightly in CO46 postvernalization, compared to a substantial decrease in Joelle. The results observed in the winter annual biotype Joelle are consistent with repression of *FLC* by vernalization. Further characterization of *FLC* at both the genome and transcriptome levels identified a one base deletion in the 5th exon coding for a keratin‐binding domain in chromosome 20 of CO46 and Joelle. The one base deletion detected in chromosome 20 *FLC* is predicted to result in a frameshift that would produce a nonfunctional protein. Analysis of whole genome sequence indicated that the one base deletion in chromosome 20 *FLC* occurred at a greater ratio in the summer biotype CO46 (2:1) compared to the winter biotype Joelle (1:4); similar trends were also observed for RNAseq and cDNA transcripts mapping to chromosome 20 *FLC* of CO46 and Joelle.

## 1 INTRODUCTION

Camelina [*Camelina sativa* (L.) Crantz] is an oilseed crop in the *Brassicaceae* family, and its genetics, production, and management have been reviewed (Berti, Gesch, Eynck, Anderson, & Cermak, 2016). Camelina has value as an industrial oilseed crop and feedstock for production of biofuels (Iskandarov, Hae, & Cahoon, 2014; Li & Mupondwa, 2014), heart‐healthy edible oil (Hines & Travis, 2016; Zubr, 2009), bio‐based pharmaceuticals and products (Horn & Benning, 2016), and as an FDA‐approved feed ingredient for chickens (Aziza, Quezada, & Cherian, 2010) and cattle (Schill, 2009). In addition to being a viable oilseed crop, some winter annual biotypes of camelina have proven winter‐hardiness traits (Gesch & Cermak, 2011). Such traits allow for their use as winter oilseed cover crops in the Great Plains and Upper Midwestern United States, where they also provide ecosystem services that include reducing erosion, scavenging and retention of nutrients, and springtime weed suppression (Berti et al., 2016), or as an early‐season nutritional source for bees (Eberle et al., 2015). The economic benefits of winter camelina as an oilseed cover crop in relay‐ and double‐cropping systems in the Upper Midwestern United States have been demonstrated (Gesch & Archer, 2013), an aspect important to the development of climate‐smart agriculture. Also, cultivars of camelina that mature earlier are desired in cover/oilseed cropping systems of northern climates, as it allows for more optimal growing conditions and full development of a second relay‐ or double‐crop [e.g., forage sorghum (*Sorghum bicolor*), sunflower (*Helianthus annuus*), and soybean [(*G.lycine max* (L.) Merr.].

Along with the model plant arabidopsis (*Arabidopsis thaliana* L.), *C. sativa* is a member of the Brassicaceae lineage I Camelineae tribe (Kagale et al., 2014). Camelina is a hexaploid (2n=40) with an estimated genome size of ~782 Mb that is believed to have originated from an allotetraploid subgenome with seven chromosomes each and a diploid subgenome with six chromosomes (Berti et al., 2016; Kagale et al., 2014). Both a reference genome sequence (Kagale et al., 2014) and a transcriptome atlas covering 88% of the annotated genes in camelina (Kagale et al., 2016) are available. Although the reference genome shares similarity to the arabidopsis genome, the hexaploid nature makes camelina more complex due to the general existence of three gene copies orthologous to arabidopsis genes. Regardless, the similar functionality and expected phenotypes of transgenic camelina expressing homologous arabidopsis genes (An & Suh, 2015; Choudhury, Riesselman, & Pandey, 2014; Lee, Kim, Kim, & Suh, 2014; Nguyen et al., 2013) and the high number of syntenically orthologous (62,277) genes (syntelogs) reported between the reference genome of *C. sativa* and *A. thaliana* suggest they likely have conserved functional characteristics (Kagale et al., 2014). Thus, it is expected that many of the camelina genes annotated to arabidopsis genes are expected to function similarly (Berti et al., 2016).

Both *C. sativa* and *A. thaliana* have a monocarpic life cycle, where plants flower only once and then die. As extensively reviewed, the vegetative to floral meristem transition in arabidopsis involves components of endogenous (age, autonomous, circadian clock, gibberellin, and sugar) and environmental (photoperiod, thermosensory, and vernalization) pathways that converge to either regulate floral integrators such as *FLOWERING LOCUS T* (*FT*), *SUPPRESSOR OF OVEREXPRESSION OF CO1* (*SOC1*), and *AGAMOUS‐LIKE24 (AGL24)*, and/or impact floral repressors such as *FLOWERING LOCUS C* (*FLC*) and *SHORT VEGETATIVE PHASE* (*SVP*); in turn, impacting various downstream meristem identity genes (Amasino, 2010; Blümel, Dally, & Jung, 2015; Henderson & Dean, 2004; Liu, Thong, & Yu, 2009; Mahrez et al., 2016; Teotia & Tang, 2015). Under ambient temperatures, FRIGIDA (FRI) is involved in activating the MADS‐box transcription factor family member *FLC*, which coincides with trimethylation of lysine 4 and 36 of histone H3 (H3K4me3 and H3K36me3), histone acetylation, and histone H2B ubiquitination (H2Bub1) of chromatin at the *FLC* loci involving Trithorax group proteins (Berry & Dean, 2015). In winter varieties of arabidopsis with functional *FLC* and *FRI*, vernalization is required to induce a vegetative to generative transition (Michaels & Amasino, 1999; Song, Angel, Howard, & Dean, 2012; Song, Shim, Kinmonth‐Schultz, & Imaizumi, 2015) that involves stable epigenetic silencing of *FLC* (Berry & Dean, 2015; Marquardt et al., 2014; Qüesta, Song, Geraldo, An, & Dean, 2016). The vernalization‐induced silencing of *FLC* (Amasino, 2010; Amasino & Michaels, 2010; Berry & Dean, 2015; Marquardt et al., 2014) is believed to involve switching *FLC*‐activating histone marks (H3K4me3/H3K36me3/H2Bub1) to *FLC* deactivating histone marks (trimethylation of histone H3 at lysine 27; H3K27me3) by a protein complex that includes polycomb repressive complex 2 (PRC2) and the plant homeodomain (PHD) family (Berry & Dean, 2015). In turn, the vernalization‐induced silencing of *FLC* inhibits its ability to repress downstream floral integrators such as *FT* and *SOC1* (Helliwell, Wood, Robertson, Peacock, & Dennis, 2006; Lee & Lee, 2010). In closely related Brassicaceae species that have a polycarpic and perennial life cycle, such as *A. halleri*,* A. lyrata,* and *Arabis alpine*, orthologues of *FLC* are also repressed by vernalization, but repression is not stable upon return to warm temperatures. Therefore, cyclic fluctuation in expression, based on seasonal environments, also plays a role in transitioning back to the vegetative state in some perennials (Aikawa, Kobayashi, Satake, Shimizu, & Kudoh, 2010; Irish, 2010; Wang et al., 2009).

Although the multiple copies of *FLC* identified in the reference genome of camelina were not reported to have obvious mutations (Kagale et al., 2014), some minor differences between paralogs of *FLC* are apparent. In other *Brassica* crops*,* such as canola (*Brassica napus L*.), up to five copies of *FLC* (*BnFLC1*‐*5*) have been identified (Tadege et al., 2001), and ~15% of the natural variation in flowering time and vernalization response in diverse accessions can be attributed to *BnFLC2* (Raman et al., 2016). In *B*. *rapa*, four copies of *FLC* have been identified and overexpression of *BrFLC2* natural antisense transcripts reduced the vernalization requirement of biennial types and reduced the growth cycle (Li, Zhang, Bai, & He, 2016). Thus, the role that each *FLC* locus plays in floral regulation remains to be determined.

The molecular processes regulating vegetative to generative development in the shoot apical meristem have not been established in camelina. Therefore, the aim of this study was to explore differences between the genotypic and phenotypic characteristics of the summer and winter flowering habits of camelina. More specifically, the objectives of this study were to (a) examine the flowering phenotypes of a summer and winter annual biotype in response to pre‐ and postvernalization treatment, (b) elucidate the transcriptome profiles of a summer and winter annual phenotype pre‐ and postvernalization, (c) characterize sequences for multiple *FLC* genes, and (d) discuss the potential involvement of other flowering time regulators.

## 2 MATERIALS AND METHODS

### 2.1 Plant growth, vernalization, and seed viability

Seeds for camelina cultivars CO46 and Joelle were obtained from the USDA‐ARS Laboratory in Morris, MN, USA, following several cycles of field production. The USDA‐ARS in Morris originally obtained the seeds from the North Dakota State University Extension Center, Carrington, ND in 2007. Seeds were sown into sunshine mix #1 (Fisons Horticulture Inc., Bellevue, WA, USA) in Deepot Cells (D60L: 6.4 cm X 35.6 cm, 983 ml volume; Stuewe & Sons, Inc., Tangent, OR, USA) and placed in support trays. Plants in each cell were thinned to a single plant at the 3–4 leaf stage. Plants were incubated in an environmental chamber for 2 weeks at 24/18°C and 16:8‐h light:dark conditions, respectively, and supplemented with water daily. At the 2‐week time point, a subset of plants were transferred to a vernalization chamber at 4°C with 8/16‐h light:dark for 4, 6, and 8 weeks (672, 1008, and 1344 chilling degree hours, respectively) and then returned to an environmental chamber set at 24/18°C 16:8 h light:dark to monitor bolting, flowering, and seed production. Stem height of individual plants, exposed to 0, 4, 6, and 8 weeks of vernalization treatment, were recorded weekly for 8 weeks posttreatment. To determine the impact of bioactive gibberellic acid (GA) on flowering, three‐week‐old glasshouse‐grown Joelle seedlings (16:8 h light:dark conditions) were treated twice weekly with or without a topical application of 100 μm GA_3_ (Sigma G7645) + 0.02% v/v Tween 20 (Sigma P‐7949) over a 6‐week period. Controls were treated with 0.02% v/v Tween 20 only.

Viability of seeds was assessed using seed silicles from the tips of 3 main stems per plant (with 20–30 dried seed/silicle). Seeds were removed from silicles and placed on 2 layers of Whatman paper moistened with 10 ml distilled water in 9 × 9 cm Petri dishes. Seeds of the winter variety Joelle were stratified at 4°C for 1 week prior to germination evaluation. Seeds were incubated at room temperature (~23°C) and germination was observed <24 hr for both varieties.

### 2.2 RNAseq

Samples collected within the upper rosette (meristematic and young leaf tissue) of individual plants exposed to 0 or 8 weeks of vernalization (pre‐ and postvernalization, respectively) were flash frozen in liquid N_2_ and stored at ‐80°C until used. Total RNA was extracted from tissue samples obtained from 3 replicate plants of CO46 and Joelle using the pine tree extraction protocol (Chang, Puryear, & Cairney, 1993) and were used to prepare RNAseq libraries for Illumina next‐generation sequencing. Stranded RNAseq libraries were prepared with Illumina's “TruSeq Stranded RNA Sample Prep Kit” with fragmentation reduced to 6 min with unique primers for each of the 12 samples (i.e., 2 camelina varieties [CO46 and Joelle] × 2 treatments [pre‐ and postvernalization] × 3 replicate plants). Libraries were pooled in equimolar concentration, and the pool was quantitated by qPCR. Sequencing was performed on one lane of a HiSeq2500 for 101 cycles from each end of the fragments using a TruSeq SBS sequencing kit version 4 at the Keck Center, Roy J. Carver Biotechnology Center, University of IL, Urbana (https://biotech.illinois.edu/htdna). Fastq files were generated and demultiplexed with the bcl2fastq v1.8.4 Conversion Software (Illumina). All sequences were deposited into NCBI BioProject ID = PRJNA292793.

### 2.3 Whole genome sequencing

DNA was extracted from leaf tissue of one CO46 and one Joelle plant using the Qiagen DNeasy Plant Maxi Kit and recommended protocol (Qiagen USA, Valencia, CA, USA). Extracted DNA was used to prepare genomic libraries with the Hyper Library Construction kit from Kapa Biosystems. Libraries were pooled in equimolar concentration, and the pool was quantitated by qPCR. Sequencing was carried out using 1 lane of a HiSeq2500 for 161 cycles from each end of the fragments using a TruSeq SBS sequencing kit version 4. Fastq files were generated and demultiplexed with the bcl2fastq v1.8.4 Conversion Software (Illumina). Sequence of adaptors used to make the libraries was as follows: Adaptor sequence in read1: AGATCGGAAGAGCACACGTCTGAACTCCAGTCACNNNNNNATCTCGTATGCCGTCTTCTGCTTG (NNNNNN= 6 nt index); and adaptor sequence in read2: AGATCGGAAGAGCGTCGTGTAGGGAAAGAGTGTAGATCTCGGTGGTCGCCGTATCAT. Average DNA fragment sizes ranged from 350 to 750 bp and reads were 160nt in length. Total reads generated for CO46 and Joelle were 183,610,576 and 169,193,270, respectively. Based on the program BBMAP, estimates of 25X coverage were predicted for conserved regions of the genome, whereas 5X coverage was predicted for the whole genome.

### 2.4 RNAseq read mapping to reference genome

Prior to read mapping, the reference genome was indexed using Bowtie2 v2.2.4 with the command “bowtie2‐build”. Trimmed and filtered RNAseq reads were mapped to the *C*. *sativa* (DH55; a doubled haploid line derived from *C*. *sativa* genotype SRS933) reference genome (Kagale et al., 2014—http://www.camelinadb.ca/downloads.html—genome version—“Cs_genome_v2.fa”, annotation file –”Cs_genes_v2_annot.gff3”) using TopHat version 2.0.13 with the following parameters: ‐G Cs_genes_v2_annot.gff3, –library‐type fr‐firststrand, ‐r 0, –mate‐std‐dev 50, ‐i 20, ‐I 20000. The output BAM file was sorted using the SAMtools (version 1.2) *sort* command. To examine stranded read mappings at the *FLC* loci, a custom set of scripts utilizing SAMtools was used to export individual BAM files for reads mapping to the forward or reverse strands <https://github.com/kevinmdorn/camelina_vernalization_rnaseq>. The stranded BAM files for each genotype were imported into CLC Genomics Workbench version 7.5.1 for visualization.

### 2.5 Whole genome sequence read mapping and analyses

Whole genome sequence (WGS) reads from Joelle and CO46 were evaluated for quality and adaptor contamination using FastQC and trimmed/filtered using BBDuk with the following parameters: ftl=10, minlen=50, qtrim=rl, trimq=10, ktrim=r, k = 25, mink=11, hdist=1, ref=/bbmap/resources/adapters.fa. Trimmed and filtered WGS reads were mapped to the *C*. *sativa* reference genome using Bowtie2 version 2.2.4 using “–sensitive‐local” setting. The output SAM file was converted to BAM format and sorted using SAMtools v1.2. The sorted BAM files were imported into CLC Genomics Workbench version 7.5.1 for visualization.

### 2.6 RT‐PCR, cloning, resequencing, and analysis

Total RNA was isolated from samples of Joelle and CO46 collected at 0, 1, 2, 4, 6, and 8 weeks of vernalization treatment. RT‐PCR was conducted using the Superscript^®^ One‐Step RT‐PCR protocol with Platinum^®^ Taq (Invitrogen, Carlsbad, CA, USA). Primers (Supporting Information Table S1) were designed using the Primer Select program of DNASTAR Lasergene 12 software (DNASTAR, Inc., Madison, WI, USA) to specifically amplify cDNA based on the assembled camelina RNAseq and WGS of CO46 and Joelle. Template RNA (1ul) was added to 1ul forward and reverse primers (at 20 pmol/ul each), 6 ul 2x Reaction Mix, and 0.5 ul Platinum^®^ Taq in a total reaction volume of 12 ul. A PTC‐200 Peltier Thermo Cycler (MJ Research, Hercules, CA, USA) was used according to the following parameters: cDNA synthesis and predenaturation, 50°C for 30 min and 94°C for 2 min; 35 cycles of PCR amplification, 94°C for 30 s to denature, 50°C for 30 s to anneal, and 72°C for 45 s to extend; 1 final extension of 72°C for 10 min; then storage at 4°C. PCR products were verified for size on agarose gels. Single bands were cut from the gels and purified using the MinElute Gel Extraction Kit (QIAGEN, Hilden, Germany). PCR products were ligated into pGEM^®^‐T Easy Vectors using the protocol for the pGEM^®^‐T Easy Vector System (Promega, Madison, WI, USA). The resulting ligation reactions were transformed into DH5α chemically competent cells (Invitrogen, Carlsbad, CA, USA) and plated on agar treated with ampicillin and IPTG and X‐gal for blue/white screening. White colonies were selected and grown overnight in 2 ml LB with ampicillin. The cultures were subsequently purified using a GeneJET Plasmid Miniprep Kit (Thermo Scientific, Vilnius, Lithuania). Purified plasmid DNA samples were sent to Iowa State University (Ames, IA, USA) for sequencing. Each clone was sequenced in both the forward and reverse directions, using the standard primers T7‐1 and SP6. Resulting sequences were trimmed of plasmid vector sequence and analyzed and aligned using DNASTAR Lasergene Software suite, version 14 (Madison, WI, USA). Gene ID abbreviations and descriptions of all putative *C. sativa* genes (Feature ID) homologous to arabidopsis (TAIR ID) included throughout this report were obtained from the arabidopsis website (www.arabidopsis.org) and are presented in Supporting Information Table S2.

### 2.7 Bioinformatic analyses

#### 2.7.1 Transcriptome assembly

RNAseq reads from the pre‐ and postvernalization experiment described above were evaluated for quality and adaptor contamination using FastQC (http://www.bioinformatics.babraham.ac.uk/projects/fastqc/). Reads were trimmed for quality and adaptor contamination using BBDuk (https://sourceforge.net/projects/bbmap/) with the following parameters: ftl=15, minlen=50, qtrim=rl, trimq=10, ktrim=r, k = 25, mink=11, hdist=1, ref=/bbmap/resources/adapters.fa, tpe tbo. Two independent transcriptome assemblies were generated with Trinity (v2.1.1) for the Joelle and CO46 genotypes using the six post‐QC RNAseq libraries from each genotype. Trinity was run using the default parameters, with the exception of “‐SS_lib_type RF” to account for the stranded RNAseq libraries.

#### 2.7.2 Identification of *FLC* chromosome 20 RNAseq fragments with a mutation

To determine the number of RNAseq fragments containing a mutation (missing T) from exon 5 of chromosome 20 *FLC*, sequence reads from CO46 and Joelle were searched using the Linux command grep for the sequences 5′‐CCATAACTAGAGCGAAGAAGACAGAACTAATGTTGAAGC‐3′, 3′‐GCTTCAACATTAGTTCTGTCTTCTTCGCTCTAGTTATGG‐5′, 5′‐CCATAACTAGAGCGAAGAAGACAGAACTAATGTGAAGC‐3′, 3′‐GCTTCACATTAGTTCTGTCTTCTTCGCTCTAGTTATGG‐5′, which are unique to this paralogue of *FLC*.

#### 2.7.3 Statistical analysis

Statistical analysis for plant growth was accomplished using a two‐stage approach as an alternative to fitting a comprehensive nonlinear mixed model (Supporting Information Data S1). This two‐stage approach first fits a Logistic growth curve to each replicate in the study and then used the resulting parameter estimates as dependent Y variables for analysis. The three parameters used in the analysis included the asymptotic height of the plant, the time at which the plant reaches half its asymptotic height, and the time elapsed from when the plant reaches half its asymptotic height to when it reaches ¾ of its asymptotic height. *R* ^2^ values for all the fitted regression curves exceeded 0.9 with most having *R* ^2^ of ≥0.99.

## 3 RESULTS

### 3.1 Phenotypic characterization

**Figure 1:**
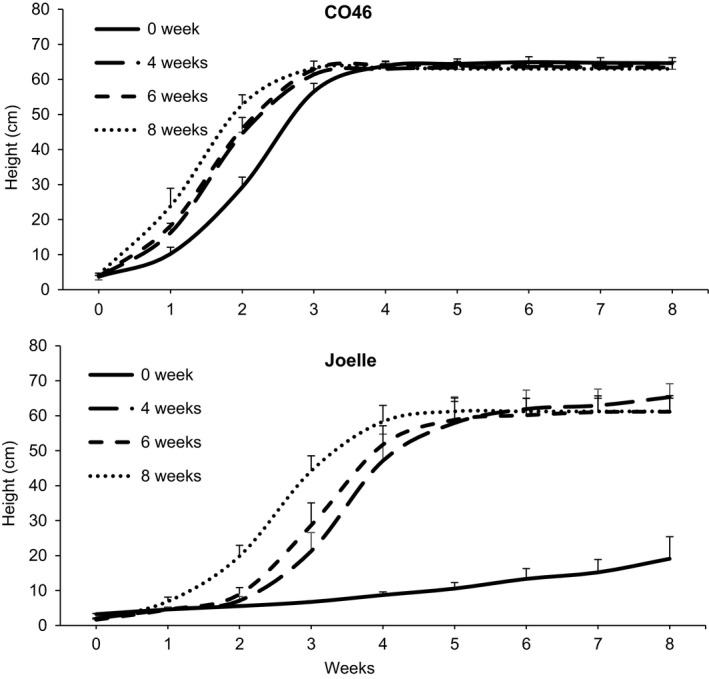
Weekly growth rate of summer (CO46) and winter (Joelle) annual biotypes of *Camelina sativa* after pre‐ (0 week) and post‐ (4, 6, and 8 week) vernalization. Plants were grown under long photoperiods (16‐hour light) pre‐ and postvernalization. Data represent three replicated studies, and each replication included three plants/sampling time point; vertical bars represent SE

Vernalization treatments (4, 6, or 8 weeks) slightly increased the bolting rate of CO46 compared to 0 weeks, whereas 4, 6, or 8 weeks vernalization all had a significant impact (Supporting Information Data S1) on the bolting rate of Joelle compared to 0 week (Figure 1). These results confirm camelina genotypes CO46 and Joelle as true summer and winter annual phenotypes, respectively, based on their requirement for vernalization‐induced bolting and flowering. The summer and winter annual phenotypes were also conserved under short‐(8 h) photoperiods (Supporting Information Figure S1), and seed produced from both varieties had 100% germination (data not presented).

### 3.2 Analyses of transcriptome profiles pre‐ and postvernalization

RNAseq data generated from replicate samples of CO46 and Joelle, pre‐ and postvernalization, produced a total of 499,156,292 reads with all libraries producing 19‐23 million reads (Supporting Information Table S3). Of the 89,416 nonredundant genes predicted in the reference genome of *C*. *sativa* (Kagale et al., 2014), assembly of RNAseq reads identified 22,157 with Fragments Per Kilobase of transcript per Million mapped reads (FPKM) values ≥5 in all three replicates for at least one of the treatments in either CO46 or Joelle, with 5,462 of those showing differential transcript abundance (tagwise dispersion based on false discovery rate [FDR] correction values of *p* < 0.05) (Table 1; Supporting Information Table S2‐CDS nonredundant genes). Based on the EDGE test for tagwise dispersion, 3,615 and 4,341 transcripts with differential abundance were identified between pre‐ and postvernalized CO46 and Joelle, respectively. Of those transcripts with FPKM≥5 and FDR≤0.05, 2,532 were common between both varieties, and 1,083 and 1,809 were unique to CO46 and Joelle, respectively. Among those transcripts that were unique, 153 and 89 transcripts had ≥2‐fold increased or decreased abundance, respectively, in CO46 postvernalization, whereas 311 and 276 transcripts had ≥2‐fold increased or decreased abundance, respectively, in Joelle postvernalization (Table 1). Among the common transcripts with differential abundance, only 13 had an opposite direction in transcript abundance.

**Table 1:** Number of annotated transcripts resulting from RNAseq analysis with FPKM≥5 in all replicates of at least one treatment, false discovery rate (FDR; *p* ≤ 0.05), with increased or decreased transcript abundance ≥2‐fold postvernalization (Post‐vern), and those that are unique or common to either summer (CO46) or winter (Joelle) annual genotypes of *Camelina sativa*

Analysis	CO46	Joelle
FPKM≥5	20656	19846
FPKM≥5 & FDR ≤0.05	3615	4341
≥2‐fold Increase Post‐vern	681	909
≥2‐fold Decrease Post‐vern	333	703
Unique ‐ FPKM≥5 & FDR ≤0.05	1083	1809
≥2‐fold Increase Post‐vern	153	311
≥2‐fold Decrease Post‐vern	89	276
Common ‐ FPKM≥5 & FDR ≤0.05	2532	
Common Increase	1157	
Common Decrease	1362	
Common Opposite	13	

### 3.3 Impact of vernalization on abundance of key transcripts involved in floral regulation pathways

In this study, transcript abundance of several classic floral regulators, such as *FT*, were minimal in both the summer and winter annual biotypes of camelina (Table 2). However, a homolog of *FT*,* TWIN SISTER OF FT* (*TSF*; Csa11 g025850), also a floral inducer, was observed to have increased transcript abundance in the summer annual genotype prevernalization. Although the increased abundance of *TSF* in a summer annual genotype is not unexpected, the increased abundance of *TFL1* (*TERMINAL FLOWER 1*), involved in the floral initiation process (generally repressing), in CO46 prior to vernalization was a surprising observation (Table 2). Regardless, the abundance for most of these floral regulators did not meet the threshold for FPKM and FDR cutoffs (Supporting Information Table S2‐CDS nonredundant genes) and, thus, the involvement of these regulators in the flowering habit of summer and winter annual biotypes of camelina will require further validation.

**Table 2:**
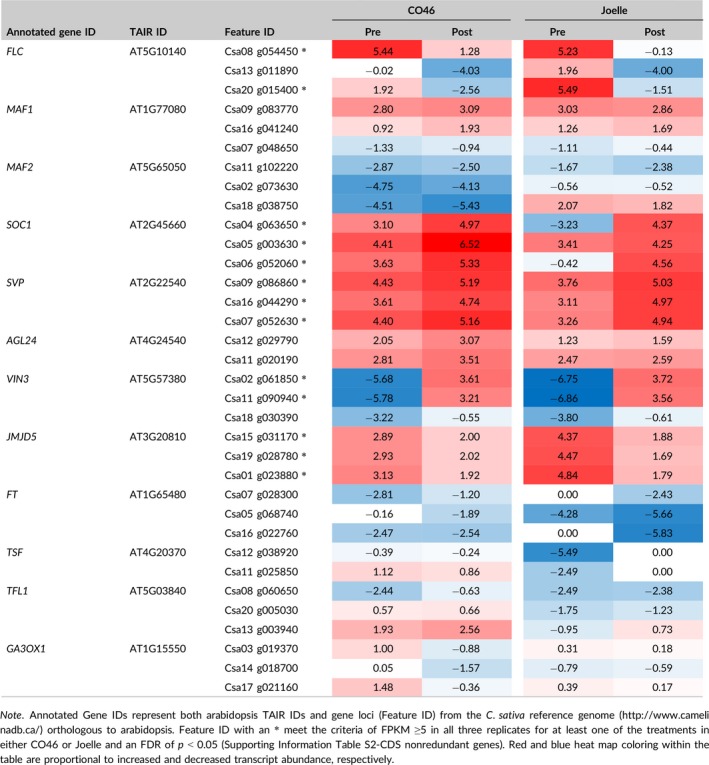
Mean FPKM (Log2) values for a summer (CO46) and winter (Joelle) annual genotype of *Camelina sativa* pre‐ and postvernalization

In arabidopsis, FLC is known to repress the floral integrator *SOC1*, whereas bioactive GA promotes flowering by upregulating *SOC1* (Conti, 2017; Helliwell et al., 2006; Lee & Lee, 2010; Moon et al., 2003). For the summer annual genotype CO46, transcript abundance of *SOC1* linked to chromosomes 4, 5, and 6 all had greater transcript abundance prevernalization compared to the winter annual genotype Joelle (Table 2; Supporting Information Table S2‐Log2 suppl). These results are consistent with increased expression of *SOC1* having a positive flowering response in the summer annual genotype CO46, independent from a vernalization treatment. In arabidopsis, SOC1 also helps to regulate *AGL24* in response to GA (Lee & Lee, 2010). Thus, the increased prevernalization abundance of *AGL24*,* SOC1* and a transcript involved in GA biosynthesis, *GA3OX1* (Table 2), in the summer annual genotype CO46 compared to the winter annual genotype Joelle would be consistent with the phenotypic responses (Figure 1) observed in this study.

**Figure 2:**
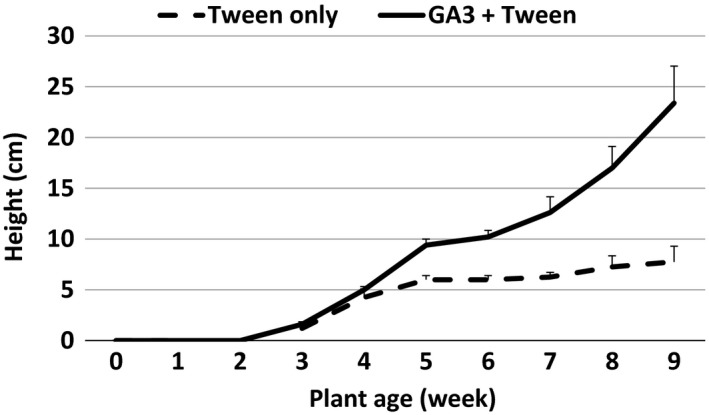
Growth response of winter annual (Joelle) *Camelina sativa* to topical application of either 0.02% v/v Tween 20 alone or 0.02% v/v Tween 20 plus 100 μm GA_3_. Five individual plants were treated twice weekly starting at 3 weeks postgermination. Data represent average mean height (cm) obtained from five individual plants, and error bars represent SE

To determine the impact of GA on inducing bolting and flowering in the winter annual biotype Joelle, we treated seedlings with topical application of bioactive GA_3_. Application of bioactive GA_3_ induced bolting in the winter annual biotype Joelle (Figure 2) without a vernalization treatment; however, it did not induce flowering. Because topical application of bioactive GA did induce bolting in the winter annual biotype without a vernalization treatment (Figure 2) but did not induce floral competence, these results support our hypothesis that altered expression or mutations to key floral regulators impacted by vernalization pathway(s) likely play a significant role in the flowering habit of camelina.

**Figure 3:**
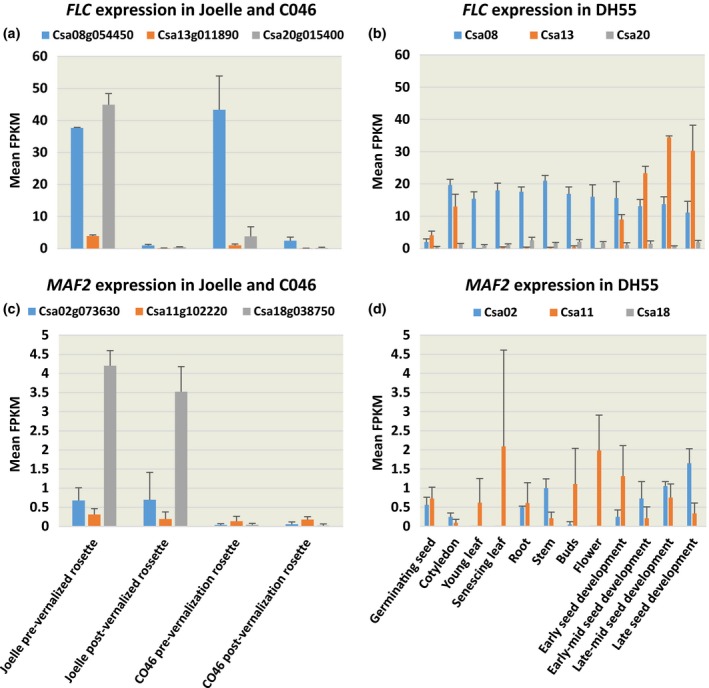
Comparison of *FLC* (a,b) and *MAF2* (c,d) transcript abundance in winter (Joelle) or summer (CO46 and DH55) annual genotypes of *Camelina sativa*. Abundance of *FLC* transcripts mapping to Csa08 g054450, Csa13 g011890, and Csa20 g015400 or *MAF2* transcripts mapping to Csa02 g073630, Csa11 g102220, and Csa18 g038750 represents mean FPKM values from rosette tissue pre‐ and postvernalization (a,c) for 8 weeks or mean FPKM values for various tissues collected from DH55 (b,d) and obtained from the publically available Camelina eFP Browser (http://bar.utoronto.ca/efp_camelina/cgi-bin/efpWeb.cgi). Error bars represent SE

The similar increase in abundance of transcript observed for *VIN3* (Table 2), a PRC component involved in vernalized repression of *FLC*, suggests that both the summer and winter genotypes of camelina respond similarly to vernalization. Indeed, vernalization of the summer and winter annual genotypes induced an overall decrease in abundance of *FLC* transcripts encoded by chromosomes 8 (Csa08 g054450), 13 (Csa13 g011890), and 20 (Csa20 g015400) (Figure 3a; Table 2; Supporting Information Table S2‐Log2 suppl). For the summer annual genotype CO46, abundance of *FLC* transcript encoded by Csa08 g054450 pre‐ and postvernalization was similar to that observed for Joelle but was greater than observed in the reference transcriptome obtained from leaf tissues of the summer genotype DH55 (Figure 3b). However, comparison of transcript abundance encoded by Csa20 g015400 in CO46 indicated it was 16‐fold less abundant than observed in Joelle prevernalization, with both CO46 and Joelle showing a decrease in transcript abundance postvernalization (Figure 3a; Table 2). Likewise, abundance of *FLC* transcript for Csa13 g011890 was greater in Joelle compared to the minimal transcript abundance in CO46 prevernalization and the transcript abundance decreased in both genotypes postvernalization. Temperature‐dependent regulation of *FLC* is also known to be moderated through jumonji demethylases (Gan et al., 2014). In this study, transcripts for all three *JUMONJI DOMAIN 5* (*JMJD5*) loci had slightly greater abundance in the winter genotype Joelle than in the summer genotype CO46 prevernalization (Table 2).

Other MADS‐box transcription factors similar to *FLC* and involved in floral regulation, such as *MAF2* (*MADS AFFECTING FLOWERING 2*), were also observed to have differential abundance between summer and winter biotypes. At low temperatures, spliced variants of *MAF1* and *MAF2* produce proteins capable of interacting with the floral repressor SVP via direct binding to the vCArG III motif in the *FT* promoter (Lee et al., 2007). Although transcript abundance varied only slightly for *SVP* in Joelle and CO46 (Table 2), all three *MAF2* orthologues (Csa02 g073630, Csa11 g102220, Csa18 g038750) did have increased transcript abundance (albeit below the 5 FPKM threshold) in Joelle relative to CO46 (Figure 3c). Interestingly, *MAF2* transcripts mapping to Csa18 g038750 were minimal in the summer genotype CO46 pre‐ and postvernalization (Figure 3c). Data extracted from the *C*. *sativa* transcriptome atlas (http://bar.utoronto.ca/efp_camelina/cgi-bin/efpWeb.cgi) also confirmed that abundance of *MAF2* transcripts mapping to Csa18 g038750 were minimal across all tissue samples tested in the summer genotype DH55.

### 3.4 Characterization of FLC

**Figure 4:**
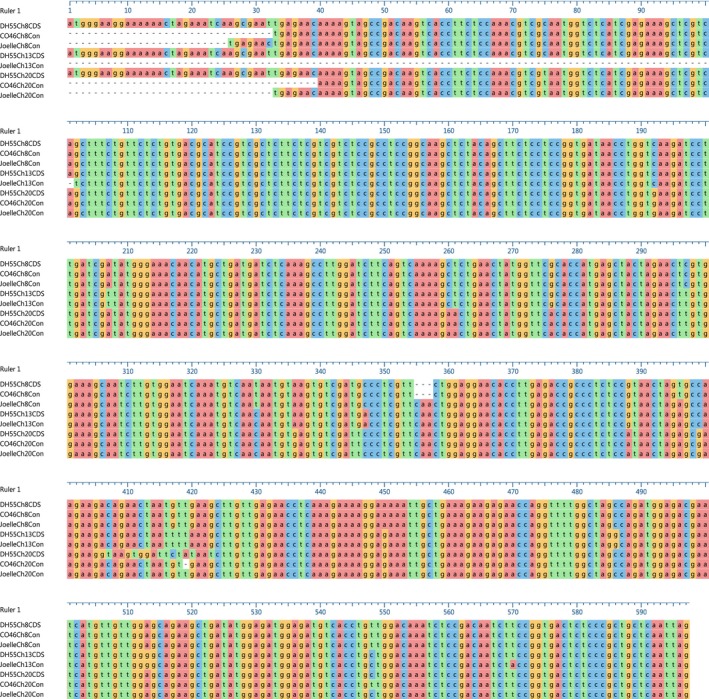
Alignment of *FLC* chromosomes 8 (Ch08), 13 (Ch13), and 20 (Ch20) sequence obtained from summer (CO46 and DH55) and winter (Joelle) annual genotypes of *Camelina sativa*. Coding sequence (CDS) was obtained from Trinity‐assembled RNAseq reads (CO46 and Joelle) or from NCBI (DH55), and consensus sequence (Cons) was obtained from sequencing of PCR‐amplified cDNA. Sequence alignments were developed using the Megalign Pro application in DNASTAR Lasergene 12 software

**Figure 5:**
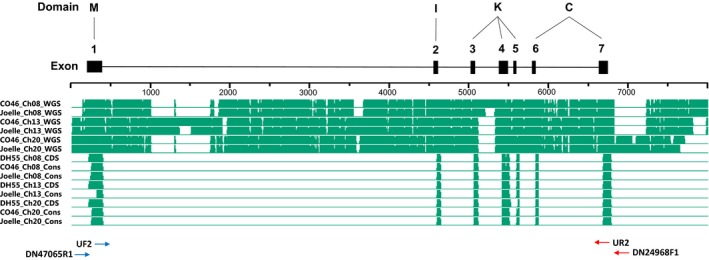
Pictograph of assembled whole genome sequence (WGS) and Trinity‐assembled RNAseq data (CDS) for *FLC* located on chromosomes 8 (Csa08), 13 (Csa13), and 20 (Csa20) obtained from the summer (CO46) or winter (Joelle) annual genotypes of *Camelina sativa*. Sequence alignments were developed using the Megalign Pro application in DNASTAR Lasergene 12 software. The full sequences corresponding to the pictograph can be viewed in Supporting Information Figure S2. The seven exons of *FLC* are outlined at the top of figure and lines connect the corresponding location of the MADS‐box (M), Intervening (I), keratin‐like (K), and C‐terminal (C) domains. Primer pairs used to amplify *FLC* for resequencing (UF2, UR2, DN47065R1, and DN24968F1) are included below the pictograph

**Figure 6:**
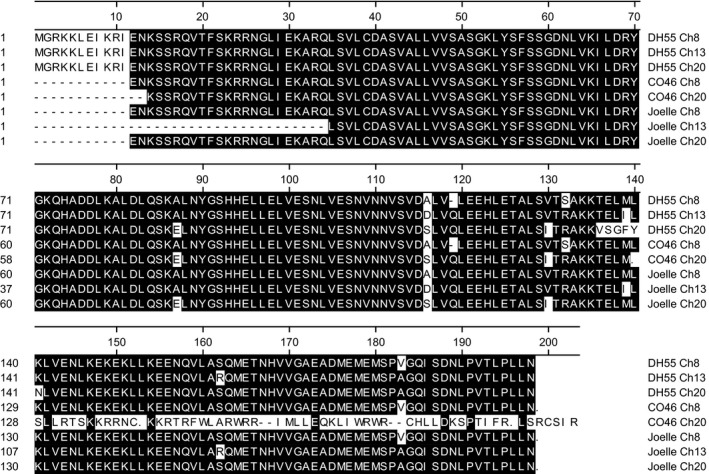
Alignment of amino acid sequence for FLC obtained from summer (CO46 and DH55) and winter (Joelle) annual genotypes of *Camelina sativa*. Sequences specific to chromosomes 8 (Ch8), 13 (Ch13), and 20 (Ch20) from the summer annual DH55 were obtained from NCBI, whereas sequence for the summer and winter annual genotypes was generated from sequence of PCR‐amplified cDNA clones. Alignments were made using the Megalign application in DNASTAR Lasergene 12 software

Whole genome sequence and coding sequence (Supporting Information Figure S2; Figure 4) for camelina *FLC* were used to develop a pictograph (Figure 5), which identifies the classic MADs‐box M‐domain for DNA binding (exon 1), an intervening I‐domain involved in protein dimerization (exon 2), a keratin‐like K‐domain for protein–protein interactions (exons 3–5), and a C‐terminal domain involved in transcriptional activation or repression (exons 6–7) (Severing et al., 2012; Li et al., 2016). Interestingly, alignment of the reference transcriptome for chromosomes 8, 13, and 20 *FLC*, obtained from DH55 (Kagale et al., 2014), indicated a significant difference in coding sequence in exon 5 of chromosome 20 (Figure 4; Supporting Information Figure S2). The 17‐base coding sequence observed at positions 405–420 of chromosome 20 in DH55 (Figure 4), corresponding to the 17 bases observed at position 5501–5517 (Supporting Information Figure S2), result in amino acid sequences at positions 136–141 (VSGFYN) of chromosome 20 in DH55 (Figure 6) being completely divergent from the amino acid sequence (TELMLK and TELILK) of chromosomes 8 and 13 in DH55, respectively. However, using a primer specific for this divergent sequence in chromosome 20 of DH55, we were not able to amplify a cDNA product using RNA isolated from either CO46 or Joelle (data not presented).

To further investigate this, we used two universal primers (Supporting Information Table S1; Figure 5) to amplify coding sequence for RNA isolated from CO46 and Joelle. Analysis of sequence from 28 randomly selected PCR‐amplified *FLC* clones from CO46 identified 27 with the best match to chromosome 8, 0 with the best match to chromosome 13, and 1 with the best match to chromosome 20. However, of 40 randomly selected PCR‐amplified *FLC* clones from Joelle, 20 had the best match to chromosome 8, 2 had the best match to chromosome 13, and 18 had the best match to chromosome 20 (Supporting Information Figure S3). The number of randomly selected cDNA from CO46 and Joelle with best sequence identity to chromosomes 8, 13, and 20 is proportional to the *FLC* transcripts identified by RNAseq prevernalization (Figure 3; Table 2). However, resequencing of PCR‐amplified *FLC* clones from either CO46 or Joelle did not identify the divergent sequence observed at position 405–420 (Figure 4) for chromosome 20 of DH55 *FLC*. Instead, all but one of the sequences obtained from Joelle, with the best match to chromosome 20 *FLC*, contained coding sequence that would produce the amino acid sequence TELMLK at position 136–141 (Figure 6). Resequencing of PCR‐amplified products did identify a 3‐base deletion (Figure 4, position 355–358) in chromosome 8 *FLC* of CO46, also observed in DH55, that codes for glutamine (Figure 6, position 119) in chromosomes 13 and 20 of CO46, DH55, and Joelle. Another surprising outcome from the resequencing of *FLC* was the identification of transcripts for chromosome 20 *FLC* that have a one base (T) deletion at position 419 (Figure 4) located within exon 5. This missing T was also observed in the assembled whole genome sequence of CO46 chromosome 20 (Supporting Information Figure S2, position 5596). This one base deletion results in a frameshift expected to produce a nonfunctional FLC protein (Figure 6) corresponding to regions K‐5 through C 6–7 of chromosome 20 in CO46 (Figure 5).

**Figure 7:**
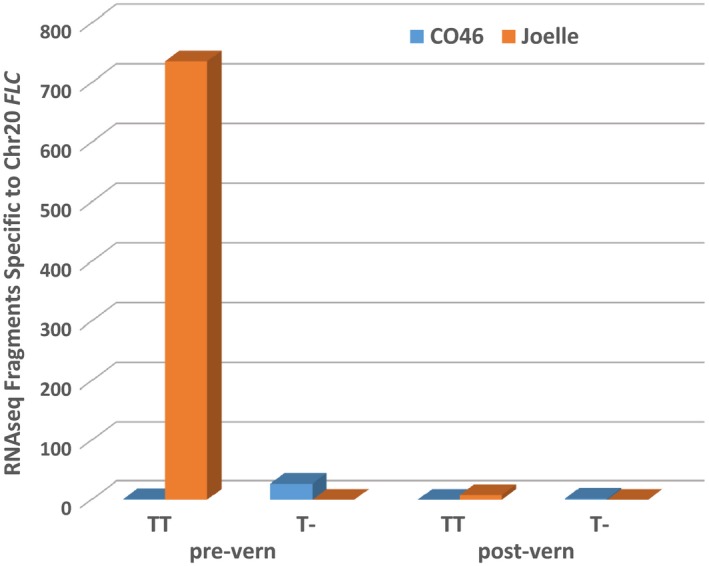
Number of RNAseq fragments identified pre‐ and postvernalization (vern) from Chr20 *FLC* in summer (CO46) or winter (Joelle) annual genotypes of *Camelina sativa* with (T‐) or without (TT) a missing T that results in a loss‐of‐function mutation

Although the assembled genome of chromosome 20 *FLC* from the winter biotype Joelle did not indicate a missing T at position 5596 (Supporting Information Figure S2), of the 18 PCR‐amplified cDNA from Joelle producing the best match to chromosome 20 *FLC*, only one (JG3Ch20C15) had a missing T (see position 386 in Supporting Information Figure S3). However, when we examined the number of fragments within our RNAseq data, which indicated 744:0 and 1:29 for a missing T (T‐) in Joelle and CO46, respectively (Figure 7), we did not observe a missing T in any of the Joelle chromosome 20 *FLC* fragments. To further investigate this phenomenon, we clustered all genomic sequence reads from CO46 and Joelle (over a 67‐base sequence covering positions 5542–5608 unique to chromosome 20 *FLC*; Supporting Information Figure S2). For the 35 genomic fragments from the summer genotype CO46 that aligned to this region, we confirmed a ratio of 2:1 for those having the one base deletion (23 reads) vs. those containing the T (12 reads), respectively, whereas, for the 26 genomic fragments from the winter genotype Joelle, the ratio was 1:4 for reads with the one base deletion (5 reads) vs. those containing the T (21 reads); see positions 158–159 in Supporting Information Figure S4. However, a chi‐square test could not rule out a 1:5 ratio (*p* = 0.72 with one degree of freedom).

## 4 DISCUSSION

The results of this study clearly indicate the existence of both summer and winter biotypes of *C*. *sativa* germplasm, based on their requirement for a vernalization treatment to induce flowering (Figure 1). The inclusion of a genomics approach also highlighted differences in some key floral regulators that likely influence camelina flowering habits (Table 2). Because a high degree of synteny and similar functionality has been observed between annotated genes of camelina and arabidopsis, their gene products are proposed to play similar roles in biological pathways and processes (Berti et al., 2016; Kagale et al., 2014). Thus, it is reasonable to speculate that one or more of the products resulting from the 3 syntenic *FLC* in summer genotypes such as CO46 likely have a mutation that does not repress *FT* or *SOC1* (Helliwell et al., 2006; Lee & Lee, 2010). Indeed, a comprehensive analysis including RNAseq, WGS, and resequencing of PCR‐amplified cDNA from the summer and winter genotypes highlighted differential abundance and mutations among the three syntenic *FLC* that could be associated with their flowering habit. The reduced abundance of *FLC* transcripts or a mutation that alters the binding of FLC to the promoter of *SOC1* (Deng et al., 2011) would both be consistent with this hypothesis and the increased transcript abundance of *SOC1* observed in the summer genotype CO46 prevernalization.

Although the results of this study focus mostly on the characteristics and association of the three syntenic camelina *FLC* loci on flowering habit, additional factors known to be associated with flowering are also discussed. For example, temperature‐dependent epigenetic regulation of *FLC* transcript abundance (Berry & Dean, 2015; Marquardt et al., 2014; McClung, Lou, Hermand, & Kim, 2016; Michaels & Amasino, 1999; Qüesta et al., 2016) and other MADS‐box transcription factors similar to FLC, such as MAF2, that are known to undergo temperature‐dependent alternative splicing, thus affecting their ability to bind to the floral repressor SVP of the thermosensory pathway (Airoldi, McKay, & Davies, 2015), are highlighted below.

### 4.1 Characterization of the three syntenic *FLC* loci

In arabidopsis, FLC can bind over 500 target sites that include promoter regions of many genes other than those involved in repression of flowering, suggesting the FLC regulates additional developmental pathways other than reproductive development (Deng et al., 2011). Based on the data presented in this study, it appears that the three camelina *FLC* loci may have distinct roles in developmental regulation during the plants’ life cycle. For example, the increased abundance of *FLC* transcripts linked to Csa20 g015400 in the winter genotype Joelle compared to summer genotypes CO46 and DH55 (Figure 3a,b) suggests it may be the most likely candidate for regulating the transition from vegetative to reproductive phase in winter biotypes. Because FLC is known to play a role in temperature‐dependent germination of arabidopsis seed (Chiang, Barua, Kramer, Amasino, & Donohue, 2009), the increased abundance of *FLC* transcript originating from the Csa13 g011890 locus (Figure 3b), in seed‐specific tissues from the summer genotype DH55 (Kagale et al., 2016), suggests it may have a more specific role during seed development and, potentially, seed germination. Finally, the increased abundance of *FLC* linked to Csa08 g054450 relative to other tissues and developmental stages in the summer genotype DH55 suggests it could have a more universal regulatory role compared to FLCs linked to chromosomes 13 and 20.

### 4.2 Factors that may affect FLC's involvement in flowering time regulation

In this study, we identified a one base (T) deletion in the genome and transcriptome of CO46 and Joelle corresponding to exon 5 of chromosome 20 *FLC*. This one base deletion is predicted to result in production of nonfunctional keratin‐binding and C‐terminal domains involved in protein–protein interactions and transcriptional activation or repression, respectively (Li et al., 2016; Severing et al., 2012). Altered expression of the K‐binding subdomains in *Brassica juncea* has been reported to interfere with homologous and heterologous interactions between FLC and SVP (Ma et al., 2015). Based on the 2:1 ratio of alleles for *FLC* on chromosome 20 in the summer annual genotype CO46, we hypothesize that there are likely 3 copies of *FLC* on chromosome 20; with 2 copies being homozygous for a one base T deletion and a third copy being homozygous for T allele (–, –, TT). In Joelle, the simplest explanation is that three copies of *FLC* exist on chromosome 20 with two being homozygous for TT and one being heterozygous (TT, TT, T‐). Although the assembled genome appears to have only a single copy of *FLC* present on chromosome 20, it is possible that tandem replicates could have been collapsed during the in silico assembly processes. Also, we certainly have not ruled out other allelic combinations or numbers of genes. However, both explanations would be consistent with the observed abundance of *FLC* alleles within our RNAseq data (Figure 7) or resequenced PCR transcripts (Supporting Information Figure S3). Thus, although the winter annual genotype Joelle has the potential to produce a small fraction of nonfunctional protein within and beyond K‐5, the increased abundance of chromosome 20 *FLC* transcripts coding for functional proteins, compared the summer annual genotype CO46, could be a factor resulting in its winter annual flowering phenotype. Another interesting divergence in predicted amino acid sequences between FLCs of the summer flowering genotypes CO46 and DH55 compared to that of winter genotype Joelle was observed for chromosome 8 where a glutamine residue was absent at position 119, and a serine for arginine substitution occurred at position 132 (Figure 6). Although it is possible these differences in amino acid sequence in chromosome 8 *FLC* impacts the summer flowering habit of camelina, this has not yet been confirmed.

### 4.3 Other factors that could play a role in the flowering habits of camelina

The cold‐dependent epigenetic switch involved in the Polycomb group silencing of *FLC* requires VAL1 localization to nucleation regions of the *FLC* genome to induce histone deacetylation and silencing of *FLC* transcription (Qüesta et al., 2016). In arabidopsis, VAL1 localizes to the RY‐1 and RY‐2 motifs within the nucleation region of intron 1 to recruit components of the PRC to shut down *FLC* transcription. Based on the assembled genomic sequence of *FLC* from summer and winter annual genotypes of camelina (Supporting Information Figure S2, see sequence positions 739–744 and 771–776) no mutations from the TGCATG sequence of predicted RY‐1 and RY‐2 motifs were observed. Because we did not see sequence differences at the VAL1 site of camelina *FLC* and many components of the PRC did not show significant differential expression between phenotypes, these seem unlikely factors to explain the resulting summer and winter phenotypes of camelina observed in this study.

Precocious flowering in arabidopsis has also been associated with temperature‐dependent regulation of *FLC* through moderation by jumonji demethylases (Gan et al., 2014). Because the histone demethylase activity of JMJD5 can remove the repressive H3k27me3 mark from the *FLC* locus at elevated temperatures in arabidopsis (Gan et al., 2014), it seems plausible to speculate that the increased abundance of *JMJD5* transcripts observed in Joelle might also influence the transcript levels of *FLC* as well. In *Medicago truncatula,* an orthologue of arabidopsis *JMJD5* has been shown to undergo cold‐dependent alternative splicing (Shen et al., 2016). Because JMJD5 is a component of the circadian clock (Jones et al., 2010; Shen et al., 2016; Yan et al., 2014), it has been suggested that cold‐dependent alternative splicing in some species might provide a link for epigenetic regulation in response to changes in circadian rhythms induced by shifts in temperature.

### 4.4 Other potential temperature‐dependent floral regulators

Noncoding‐ and long noncoding RNAs (lncRNAs) that are antisense components of arabidopsis *FLC* (Csorba, Questa, Sun, & Dean, 2014; Swiezewski, Liu, Magusin, & Dean, 2009) are collectively referred to as COOLAIR (cold‐induced long antisense intragenic RNA). Prolonged cold induces expression of *COOLAIR*, which in turn physically binds to *FLC* chromatin nucleation sites to induce epigenetic silencing of *FLC* as previously described for demethylation of the *FLC*‐activating histone marks (H3k4me3 and H3K36me3) (Berry & Dean, 2015; Csorba et al., 2014; Letswaart, Wu, & Dean, 2012; Qüesta et al., 2016). However, alternative splicing of *COOLAIR* in arabidopsis impacts the cotranscriptional coupling mechanisms that affect *FLC* expression (Marquardt et al., 2014).

In other MADS‐box transcription factors similar to *FLC*, such as *MAF1* and *MAF2*, temperature‐dependent alternative splicing also impacts their ability to interact with the floral repressor SVP within the thermosensory pathway (Airoldi et al., 2015). When ambient temperatures increase, altered splicing produces MAF1 and MAF2 variants incapable of interacting with SVP to suppress flowering. In this study, all three *MAF2* orthologues did have increased transcript abundance (albeit below the 5 FPKM threshold) in Joelle relative to CO46. The minimal abundance of transcripts mapping to Csa18 g038750 observed in summer genotypes CO46 pre‐ and postvernalization and in DH55 could suggest that the *MAF2* loci on *C*. *sativa* chromosome 18 play some role in the winter annual life cycle. However, because we did not quantify transcript abundance after returning vernalized plants to growth conducive conditions, further research is warranted to determine the effects that ambient temperatures have on these MADS‐box transcription factors during bolting and floral development postvernalization.

## 5 CONCLUSIONS

This study confirmed the existence of a true winter cultivar of camelina based on the requirement for a floral‐inducing vernalization treatment. Although a mutation discovered in the genome and transcriptome of chromosome 20 *FLC* is predicted to result in a nonfunction protein that would likely impact its ability to repress floral integrators in summer biotypes of camelina, further studies are needed to functionally confirm this theory. Transcriptome analyses also confirmed increased abundance of MADS‐box transcription factors similar to *FLC*, such as *MAF2*, in the winter annual genotype that might also influence flowering habits. Because *MAF2* can undergo temperature‐dependent splicing to influence the ability of MAF2 to interact with other floral regulators in arabidopsis, the similar expression levels observed pre‐ and postvernalization for *MAF2* in the winter annual genotype do not necessarily predict the functional activity of MAF2. However, the involvement of antisense transcripts of *FLC*, such as *COOLAIR* and *COLDAIR*, and the impact of temperature‐dependent alternative splicing of *MAF2* on flower habit in camelina are beyond the focus of this study and will require further research.

## AUTHORS‘CONTRIBUTIONS

JVA, RG, and MDM conceived and designed the experiments. JVA, AGH, and EEW performed the experiments. JVA, KMD, AGH, DPH, WSC, and MD analyzed the data. JVA, DPH, WSC, and MD wrote the manuscript, and all authors revised and approved the final manuscript.
