# Supplementary figures and images for: Expression of FLOWERING LOCUS C and a frameshift mutation of this gene on chromosome 20 differentiate a summer and winter annual biotype of Camelina sativa
Source: Plant Direct. 2018 Jul 9;2(7):e00060. doi: 10.1002/pld3.60 (PMC6508819; doi:10.1002/pld3.60)

Supplemental Figure 1.

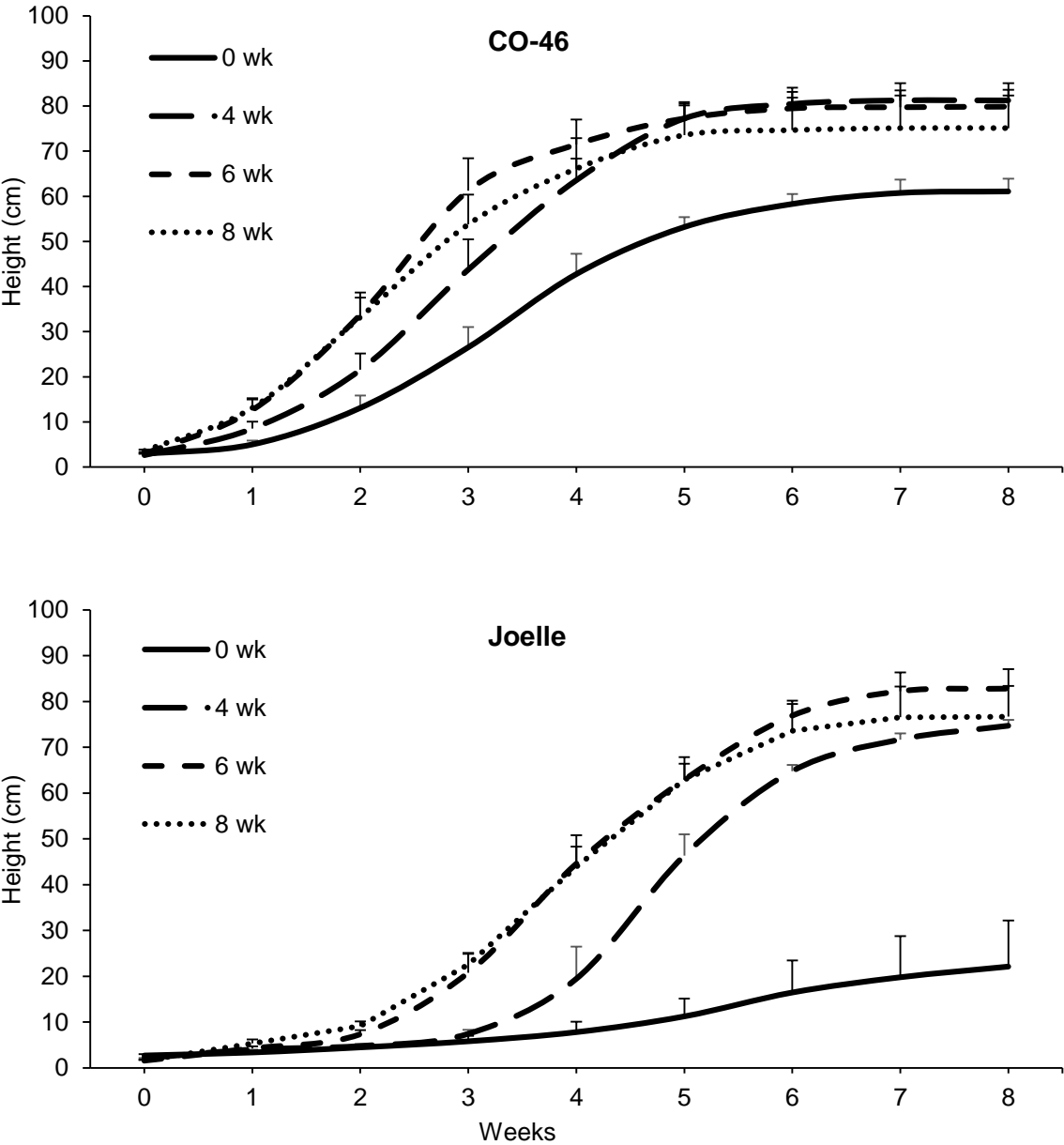

Supplement: Supplementary file 1 [file PLD3-2-e00060-s001.pdf]

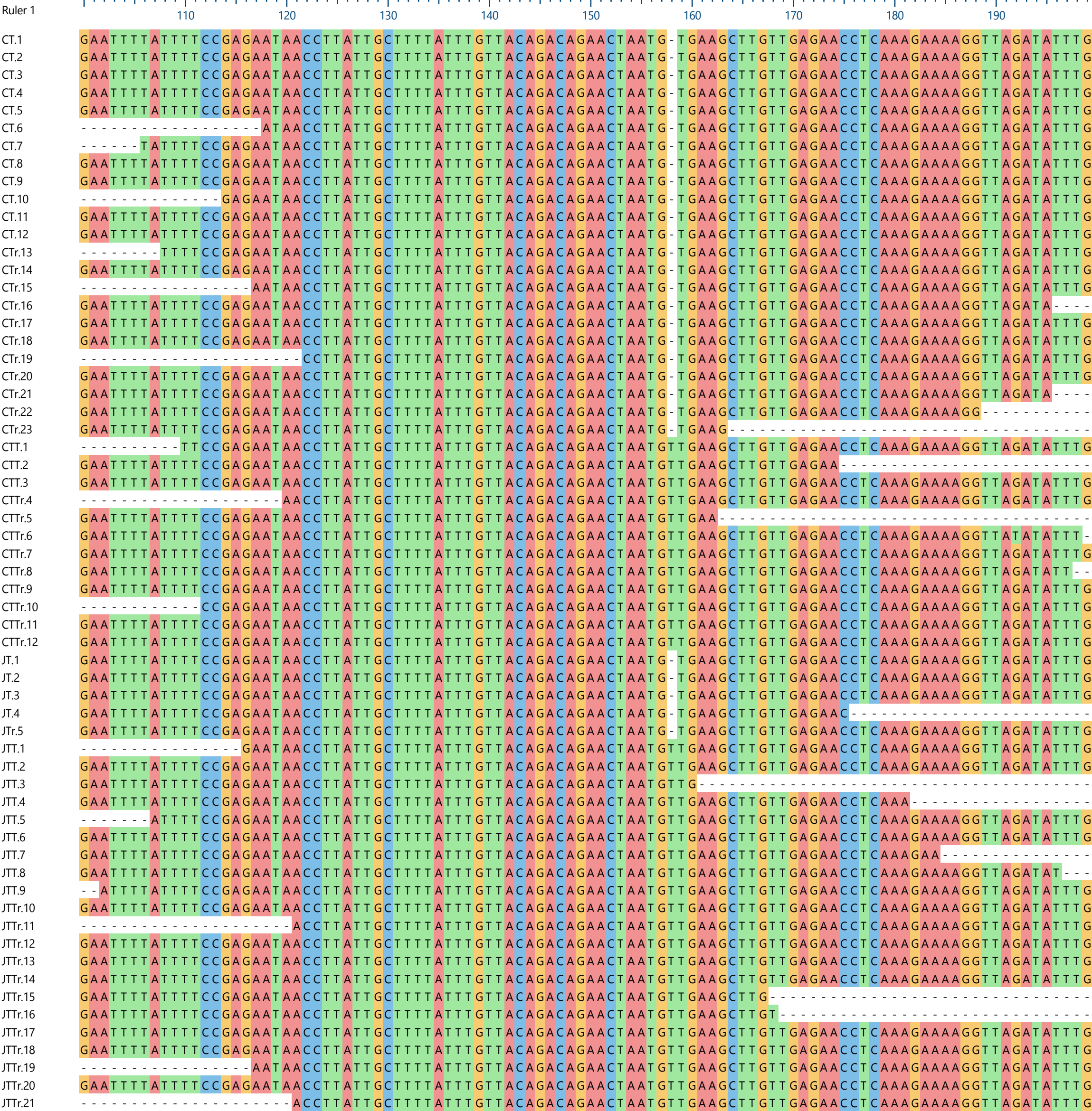

Supplement: Supplementary file 4 [file PLD3-2-e00060-s004.pdf]
