# Supplementary material for: Expression of FLOWERING LOCUS C and a frameshift mutation of this gene on chromosome 20 differentiate a summer and winter annual biotype of Camelina sativa
Source: Plant Direct. 2018 Jul 9;2(7):e00060. doi: 10.1002/pld3.60 (PMC6508819; doi:10.1002/pld3.60)
Supplement: Supplementary file 3 [file PLD3-2-e00060-s003.pdf]

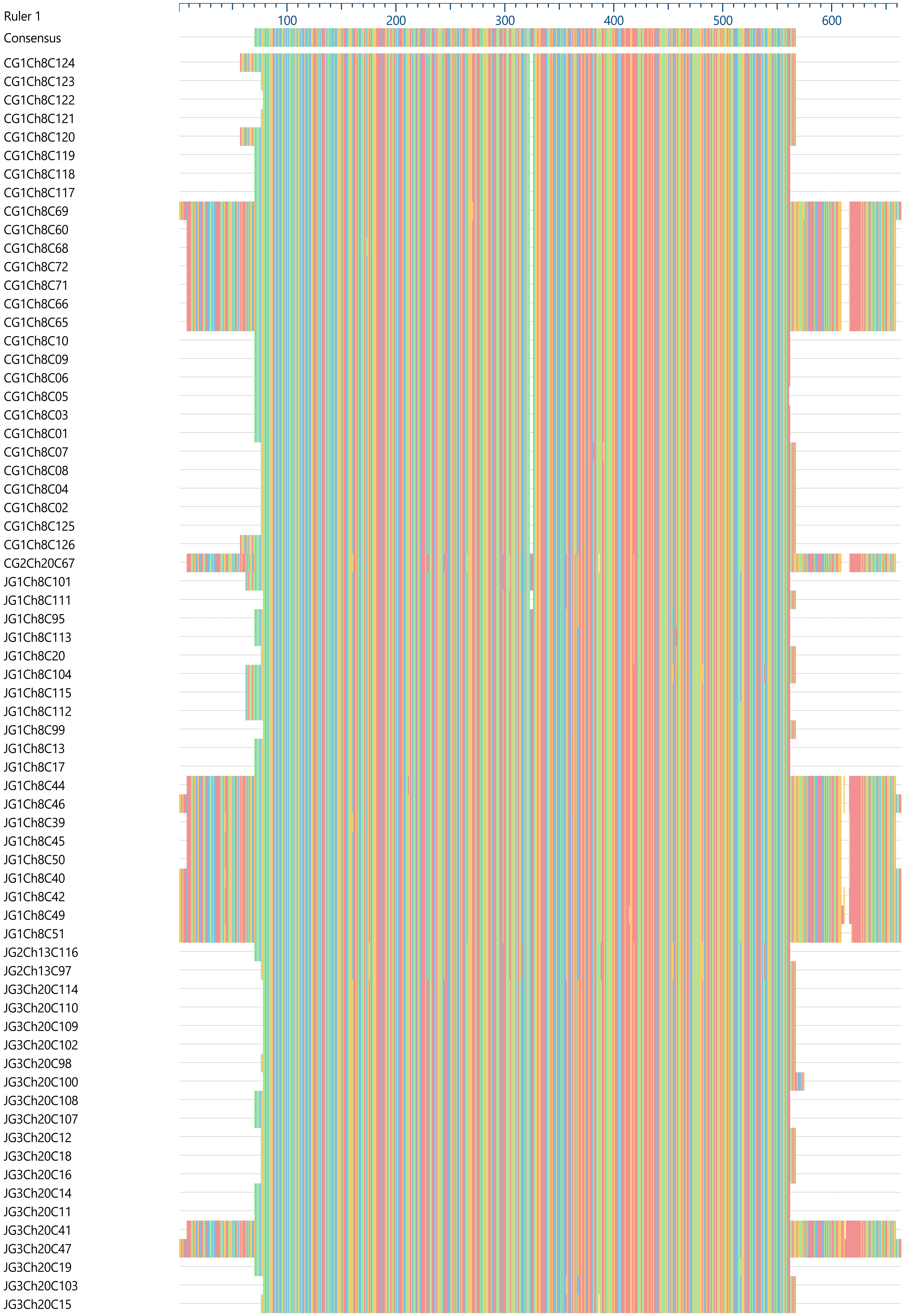
Ruler 1
100
200
300
400
500
600
Consensus
CG1Ch8C124
CG1Ch8C123
CG1Ch8C122
CG1Ch8C121
CG1Ch8C120
CG1Ch8C119
CG1Ch8C118
CG1Ch8C117
CG1Ch8C69
CG1Ch8C60
CG1Ch8C68
CG1Ch8C72
CG1Ch8C71
CG1Ch8C66
CG1Ch8C65
CG1Ch8C10
CG1Ch8C09
CG1Ch8C06
CG1Ch8C05
CG1Ch8C03
CG1Ch8C01
CG1Ch8C07
CG1Ch8C08
CG1Ch8C04
CG1Ch8C02
CG1Ch8C125
CG1Ch8C126
CG2Ch20C67
JG1Ch8C101
JG1Ch8C111
JG1Ch8C95
JG1Ch8C113
JG1Ch8C20
JG1Ch8C104
JG1Ch8C115
JG1Ch8C112
JG1Ch8C99
JG1Ch8C13
JG1Ch8C17
JG1Ch8C44
JG1Ch8C46
JG1Ch8C39
JG1Ch8C45
JG1Ch8C50
JG1Ch8C40
JG1Ch8C42
JG1Ch8C49
JG1Ch8C51
JG2Ch13C116
JG2Ch13C97
JG3Ch20C114
JG3Ch20C110
JG3Ch20C109
JG3Ch20C102
JG3Ch20C98
JG3Ch20C100
JG3Ch20C108
JG3Ch20C107
JG3Ch20C12
JG3Ch20C18
JG3Ch20C16
JG3Ch20C14
JG3Ch20C11
JG3Ch20C41
JG3Ch20C47
JG3Ch20C19
JG3Ch20C103
JG3Ch20C15
