# Supplementary material for: Expression of FLOWERING LOCUS C and a frameshift mutation of this gene on chromosome 20 differentiate a summer and winter annual biotype of Camelina sativa
Source: Plant Direct. 2018 Jul 9;2(7):e00060. doi: 10.1002/pld3.60 (PMC6508819; doi:10.1002/pld3.60)
Supplement: Supplementary file 5 [file PLD3-2-e00060-s005.pdf]

Supplementary Table 1. Primers used for PCR amplification of *Flowering Locus C* cDNA from a

**Primer name**	**GENE ID ABV.**	5'F/R	Primer Sequence
FLC-UF2	FLC	5'F	CTT TCT GTT CTC TGT GAC GCA TCC
FLC-UR2	FLC	5'R	CTA ATT GAG CAG CGG GAG AGT
DN24968F1	FLC	5'F	TAC GAC GCG CCC TTA TC
DN47065R1	FLC	5'R	TGA GAA CAA AAG TAG CCG ACA AG
