# Supplementary material for: Expression of FLOWERING LOCUS C and a frameshift mutation of this gene on chromosome 20 differentiate a summer and winter annual biotype of Camelina sativa
Source: Plant Direct. 2018 Jul 9;2(7):e00060. doi: 10.1002/pld3.60 (PMC6508819; doi:10.1002/pld3.60)
Supplement: Supplementary file 7 [file PLD3-2-e00060-s007.pdf]

Supplementary Table 3. Number of RNAseq reads obtained from 12 libraries. Libraries were constructed from RNA isolated from 3 replicate tissue samples from a winter- (Joelle) or summer- (CO46) annual genotype of *C. sativa* with (post-) or without (pre-) veralization treatment.

	Variety	ID	Name of Fastq (sampleID_index_lane#_read#_001.fastq)	Number of Reads
pre-vernalization	Joelle	Cs01	Cs01_ATCACG_L006_R1_001.fastq	20,134,301
	Joelle		Cs01_ATCACG_L006_R2_001.fastq	20,134,301
	Joelle	Cs02	Cs02_CGATGT_L006_R1_001.fastq	21,821,142
	Joelle		Cs02_CGATGT_L006_R2_001.fastq	21,821,142
	Joelle	Cs03	Cs03_TTAGGC_L006_R1_001.fastq	21,581,508
	Joelle		Cs03_TTAGGC_L006_R2_001.fastq	21,581,508
	CO-46	Cs04	Cs04_TGACCA_L006_R1_001.fastq	19,803,900
	CO-46		Cs04_TGACCA_L006_R2_001.fastq	19,803,900
	CO-46	Cs05	Cs05_ACAGTG_L006_R1_001.fastq	20,205,021
	CO-46		Cs05_ACAGTG_L006_R2_001.fastq	20,205,021
	CO-46	Cs06	Cs06_GCCAAT_L006_R1_001.fastq	20,650,549
	CO-46		Cs06_GCCAAT_L006_R2_001.fastq	20,650,549
Post-vernalization	Joelle	Cs07	Cs07_CAGATC_L006_R1_001.fastq	21,220,464
	Joelle		Cs07_CAGATC_L006_R2_001.fastq	21,220,464
	Joelle	Cs08	Cs08_ACTTGA_L006_R1_001.fastq	20,811,901
	Joelle		Cs08_ACTTGA_L006_R2_001.fastq	20,811,901
	Joelle	Cs09	Cs09_GATCAG_L006_R1_001.fastq	19,257,186
	Joelle		Cs09_GATCAG_L006_R2_001.fastq	19,257,186
	CO-46	Cs10	Cs10_TAGCTT_L006_R1_001.fastq	21,485,460
	CO-46		Cs10_TAGCTT_L006_R2_001.fastq	21,485,460
	CO-46	Cs11	Cs11_GGCTAC_L006_R1_001.fastq	19,471,177
	CO-46		Cs11_GGCTAC_L006_R2_001.fastq	19,471,177
	CO-46	Cs12	Cs12_CTTGTA_L006_R1_001.fastq	23,135,537
	CO-46		Cs12_CTTGTA_L006_R2_001.fastq	23,135,537
			**Total Reads:**	**499,156,292**
