# Supplementary material for: Expression of FLOWERING LOCUS C and a frameshift mutation of this gene on chromosome 20 differentiate a summer and winter annual biotype of Camelina sativa
Source: Plant Direct. 2018 Jul 9;2(7):e00060. doi: 10.1002/pld3.60 (PMC6508819; doi:10.1002/pld3.60)
Supplement: Supplementary file 8 [file PLD3-2-e00060-s008.pdf]

Supplementary Material 1. Analysis using a two stage approach

As an alternative to fitting a comprehensive nonlinear mixed model this approach first fits a Logistic growth curve to each replicate in the study and uses the resulting parameter estimates as dependent Y variables for analysis. As before there are three parameters:

ϕ_1 for the asymptotic height of the plant. This paramer is labeled Asym in analyses.

ϕ_2 gives the time at which the plant reaches half its asymptotic height. This parameter is labelled xmid in analyses.

ϕ_3 gives the time elapsed for a plant from lfrom the time it reaches half its asymptotic height to when it reaches about 3/4 of its asyptotic height .

R^2 for all of the fitted regression curves exceeded 0.9 with most having R^2 of 0.99 or more.

A plot of the parameter estimates with their corresponding line and treatments is given below.

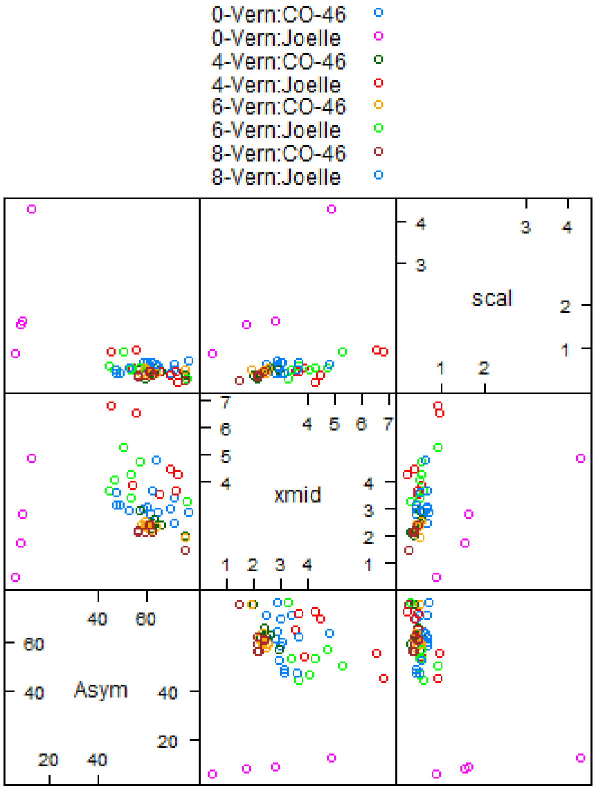

Scatter Plot Matrix

The plot shows the growth curves for the Joelle line under the 0 vernilization to be much different than the growth curves for the other treatment/line combinations. This curve has lower asymptotic growth (Asym values are lower), a longer time between half its asymptotic growth and 3/4 its asymptotic growth (scal tends to be larger).

The parameter estimates for each of the reps were then saved to a dataset with their corresponding treatment and line and these were analyzed using anova methods.

Analysis was conducted on a per parameter basis.

For the asymptote (Asym) we get the following:

```
Analysis of Variance Table of type III  with  Satterthwaite
approximation for degrees of freedom
               Sum Sq Mean Sq NumDF DenDF F.value  Pr(>F)
Treatment        4123    1374     3    43    44.3 3.3e-13 ***
Line             3750    3750     1    43   120.9 4.6e-14 ***
Treatment:Line   5068    1689     3    43    54.4 1.1e-14 ***
---
Signif. codes:  0 '***' 0.001 '**' 0.01 '*' 0.05 '.' 0.1 ' ' 1


Line = CO-46:
 Treatment lsmean   SE   df lower.CL upper.CL .group
 8-Vern      64.5 5.72 2.56    44.36     84.6  a
 6-Vern      65.3 5.72 2.56    45.20     85.5  a
 4-Vern      66.3 5.72 2.56    46.20     86.5  a
 0-Vern      68.0 5.72 2.56    47.92     88.2  a
Line = Joelle:
 Treatment lsmean   SE   df lower.CL upper.CL .group
 0-Vern      13.5 6.05 3.19    -5.13     32.2  a
 6-Vern      57.3 5.72 2.56    37.14     77.4   b
 8-Vern      59.9 5.72 2.56    39.77     80.0   b
 4-Vern      64.8 5.72 2.56    44.68     84.9   b
Confidence level used: 0.95
P value adjustment: tukey method for comparing a family of 4 estimates
significance level used: alpha = 0.05

 Line   lsmean   SE   df lower.CL upper.CL .group
 Joelle   48.9 5.45 2.11     26.5     71.2  a
 CO-46    66.0 5.43 2.07     43.4     88.7   b
Results are averaged over the levels of: Treatment
Confidence level used: 0.95
significance level used: alpha = 0.05
```

For the xmid parameter we get the following:

```
Analysis of Variance Table of type III  with  Satterthwaite
approximation for degrees of freedom
               Sum Sq Mean Sq NumDF DenDF F.value  Pr(>F)
Treatment        6.14    2.05     3  42.7     3.4 0.02676 *
Line            19.83   19.83     1  42.8    32.7 9.5e-07 ***
Treatment:Line  12.22    4.07     3  42.7     6.7 0.00082 ***
---
Signif. codes:  0 '***' 0.001 '**' 0.01 '*' 0.05 '.' 0.1 ' ' 1

Line = CO-46:
 Treatment lsmean    SE   df lower.CL upper.CL .group
```

```
 8-Vern      2.07 0.342 13.3     1.33     2.80  a
 6-Vern      2.25 0.342 13.3     1.51     2.99  a
 4-Vern      2.34 0.342 13.3     1.60     3.08  a
 0-Vern      2.88 0.342 13.3     2.14     3.61  a
Line = Joelle:
 Treatment lsmean    SE   df lower.CL upper.CL .group
 0-Vern      2.42 0.443 23.2     1.51     3.34  a
 8-Vern      3.39 0.342 13.3     2.65     4.12  ab
 6-Vern      4.04 0.342 13.3     3.30     4.78   bc
 4-Vern      4.68 0.342 13.3     3.94     5.42    c
Confidence level used: 0.95
P value adjustment: tukey method for comparing a family of 4 estimates
significance level used: alpha = 0.05

 Line   lsmean    SE   df lower.CL upper.CL .group
 CO-46    2.38 0.228 2.85     1.64     3.13  a
 Joelle   3.63 0.241 3.34     2.91     4.36   b
Results are averaged over the levels of: Treatment
Confidence level used: 0.95
significance level used: alpha = 0.05
```

Finally for the scal parameter we get:

```
Analysis of Variance Table of type III  with  Satterthwaite
approximation for degrees of freedom
               Sum Sq Mean Sq NumDF DenDF F.value  Pr(>F)
Treatment        5.81    1.94     3    45   11.17 1.3e-05 ***
Line             2.97    2.97     1    45   17.13 0.00015 ***
Treatment:Line   4.05    1.35     3    45    7.78 0.00027 ***
---
Signif. codes:  0 '***' 0.001 '**' 0.01 '*' 0.05 '.' 0.1 ' ' 1

Line = CO-46:
 Treatment lsmean    SE   df lower.CL upper.CL .group
 8-Vern     0.351 0.159 34.0   0.0275    0.675  a
 4-Vern     0.415 0.159 34.0   0.0909    0.738  a
 6-Vern     0.430 0.159 34.0   0.1066    0.754  a
 0-Vern     0.538 0.159 34.0   0.2144    0.862  a
Line = Joelle:
 Treatment lsmean    SE   df lower.CL upper.CL .group
 6-Vern     0.522 0.159 34.0   0.1979    0.845  a
 8-Vern     0.531 0.159 34.0   0.2077    0.855  a
 4-Vern     0.536 0.159 34.0   0.2121    0.860  a
 0-Vern     2.072 0.221 35.8   1.6249    2.520   b
Confidence level used: 0.95
P value adjustment: tukey method for comparing a family of 4 estimates
significance level used: alpha = 0.05

 Line   lsmean     SE   df lower.CL upper.CL .group
 CO-46   0.434 0.0824 4.88     0.22    0.647  a
 Joelle  0.915 0.0917 5.87     0.69    1.141   b
Results are averaged over the levels of: Treatment
Confidence level used: 0.95
significance level used: alpha = 0.05
```
