# Supplementary material for: Expression of FLOWERING LOCUS C and a frameshift mutation of this gene on chromosome 20 differentiate a summer and winter annual biotype of Camelina sativa
Source: Plant Direct. 2018 Jul 9;2(7):e00060. doi: 10.1002/pld3.60 (PMC6508819; doi:10.1002/pld3.60)
Supplement: Supplementary file 9 [file PLD3-2-e00060-s009.pdf]

MSID: 2018-00144

MS TITLE: Expression of *Flowering Locus C* and a frame shift mutation of this gene on Chromosome 20 differentiate a summer- and winter-annual biotype of *Camelina sativa*

Specific response to Reviewer #1, Reviewer #2, and the editor are listed below on a point-by-point basis in blue highlighting. Corresponding changes within the text of the manuscript are also highlighted in blue. The authors thank the reviewers and editor of their helpful comments, insights, and suggestions, which helped to improve the overall quality of the manuscript.

Reviewer #1

The manuscript by Anderson et al., titled "Expression of Flowering Locus C and a frame shift mutation of this gene on Chromosome 20 differentiate a summer- and winter-annual biotype of *Camelina sativa*" is well written and makes a compelling, scientifically sound case for *FLC* playing an important role in the expression of either the summer- or winter-annual phenotype in camelina, as supported by the differences in transcript abundance of the *FLC* orthologue mapping to chromosome 20 pre-vernalization and the identification of a single base pair deletion in this gene causing a frame shift leading to a non-functioning protein.

I would like to make the following suggestions that may help to improve the manuscript:

Abstract:

"… (FLC; Csa20g258400) was 16-fold greater in Joelle…: Although it is mentioned in the Result section that this gene was markedly less abundant in CO46 than in Joelle pre-vernalization, this number is not mentioned. I suggest including this number also in the Results section. Done

"… compared to a substantial and significant decrease in Joelle." This is saying things twice over, as one assumes that anything that is substantial is also significant. I suggest to delete "and significant". Done

"However, further characterization of FLC at both the genome and transcriptome levels …" I don't see the necessity of the word "However" at the beginning of the sentence as the presented results don't contradict each other; therefore, I suggest to delete "however" and start the sentence just with "Further". Done

Introduction:

"…, and FDA approved feed supplement for chickens …" Camelina meal is not approved as a supplement but an ingredient; therefore please change to "… feed ingredient". Done

"Such traits allow for their use as a winter oilseed cover crop in cold regions, …" What do you mean by "cold regions"? Changed to read "...in the Great Plains and Upper Midwestern United States,..."

"However, cultivars of camelina that have an earlier maturity trait are desired …". Winter camelina doesn't necessarily have an earlier maturity trait. They do mature earlier because they have been seeded in the fall and established a rosette before winter. Suggestion: "Also, cultivars of camelina that mature earlier in the season are desired…" Changed to read as suggested

"....the model plant Arabidopsis (Arabidopsis Thaliana L.) Changed "T" to "t"

"… and the high degree of syntenically orthologous…" Saying "degree" when referring to an absolute number is not correct; Suggestion: The high **number** of syntenically orthologous …." Done

In the Introduction section, there is a focus on the genes *FLC* and *FRI*. While this makes sense for *FLC*, the gene FRI however is not mentioned once again in either Result or Discussion. I suggest keeping the mention of *FRI* to a minimum (necessary to explain the context of the study). Particularly in the sentence "The multiple copies of FLC and FRI identified …", the mention of FRI is unnecessary. Sentence in introduction changed to read "…multiple copies of *FLC* identified in…"

It might make sense to also mention the other genes that were investigated at the top of page 4, where the authors talk about the examples of different floral integrators and floral repressors. Additional information was added to result section, where appropriate.

Materials and Methods:

Plant growth, …:

CO46 is a camelina variety that was developed by Sustainable Oils. Even though the company doesn't exist anymore, the breeder should be given credit. For Joelle on the other hand, it seems impossible to find out who originally developed it. First sentence of M&M revised to read " Seeds for camelina cultivars CO46 and Joelle, were obtained from the USDA-ARS Laboratory in Morris, MN, USA following several cycles of field production. The USDA-ARS in Morris originally obtained the seeds from the North Dakota State University Extension Center, Carrington, ND in 2007. Seeds…"

"However, seeds of the winter variety…". I suggest to delete the word "however". Done

RNAseq analysis: This paragraph doesn't include the description of an analysis; therefore, I would change the heading to just *RNAseq* or *RNA sequencing*. Changed to "*RNAseq*" as suggested

Whole genome sequencing: How many plants did you collect tissue from for each genotype? Changed to read "DNA was extracted from leaf tissue of one CO46 and one Joelle plant using…"

I suggest putting "Bioinformatics analyses" after "RT-PCR, cloning, sequencing and analysis" and before "Statistical analysis". I also suggest to change the heading "RT-PCR, cloning, sequencing and analysis" to "RT-PCR, cloning, **re**sequencing and analysis" Done, as suggested.

The following was also added to the end of the **Bioinformatics analyses** section:

"***Identification of FLC chromosome 20 RNAseq fragments with a mutation***

To determine the number of RNAseq fragments containing a mutation (missing T) from exon 5 of chromosome 20 *FLC*, sequence reads from CO46 and Joelle were searched using the Linux command grep for the sequences 5'-CCATAACTAGAGCGAAGAAGACAGAACTAATGTTGAAGC-3', 3'-GCTTCAACATTAGTTCTGTCTTCTTCGCTCTAGTTATGG-5', 5'-CCATAACTAGAGCGAAGAAGACAGAACTAATGTGAAGC-3', 3'-GCTTCACATTAGTTCTGTCTTCTTCGCTCTAGTTATGG-5', which are unique to this paralogue of *FLC*."

"*RT-PCR, cloning, sequencing and analysis*": Typo in Vinius: Vi**l**nius. Changed to "Vilnius"

Results:

Results regarding *FLC* expression of DH55, which is shown in Figure 3B, are not described in the text but discussed later. The following was added to the sentence including first mention of Fig. 3 in the results section: "For the summer annual genotype CO46, abundance of *FLC* transcript encoded by Csa08g054450 pre- and post-vernalization was similar to that observed for Joelle but was greater than observed in the reference transcriptome obtained from leaf tissues of the summer biotype DH55 (Fig. 3B)."

"…transcripts mapping to Csa18g038750 were minimal across all tissues samples tested …" Please change to "tissues sampled" or "tissue samples". Also, refer to Figure 3D after this sentence ("…tested in the summer genotype DH55 (fig. 3D)." Done

"These results could suggest that the *MAF2* loci on *C. sativa* chromosome 18 has some role in the winter annual life cycle". This is an interpretation of the results and should go into the Discussion section (where it also is). This sentence was removed from the results: "~~These results could suggest that the *MAF2* loci on *C. sativa* chromosome 18 has some role in the winter annual life cycle.~~"

",… whereas, for 26 genomic fragments from the winter genotype Joelle the ratio was 1:4 for reads containing the T (21 reads) vs. those with the one base deletion (5 reads)." For this to be less confusing, it should read "for 26 genomic fragments from the winter genotype Joelle the ratio was 1:4 for reads with the one base deletion (5 reads) vs. those containing the T (21 reads)." Then, in the last sentence, it should read "a Chi square test could not rule out a **1:5** ratio…".
The last paragraph of the results section has been revised to read "Although the assembled genome of chromosome 20 *FLC* from Joelle did not indicate a missing T at position 5596 (Supplementary Fig. 2), of the 18 PCR amplified cDNA from Joelle producing the best match to chromosome 20 *FLC*, only one (JG3Ch20C15) had a missing T (see position 386 in Supplementary Fig. 3). However, when we examined the number of fragments within our RNAseq data, which indicated 744:0 and 1:29 for a missing T (T-) in Joelle and CO46, respectively (Fig. 7), we did not observe a missing T in any of the Joelle chromosome 20 *FLC* fragments. To further investigate this phenomenon, we clustered all genomic sequence reads from CO46 and Joelle (over a 67-base sequence covering positions 5542-5608 unique to chromosome 20 *FLC*; Supplementary Fig. 2). For the 35 genomic fragments from the summer genotype CO46 that aligned to this region, we confirmed a ratio of 2:1 for those having the one base deletion (23 reads) vs. those containing the T (12 reads), respectively, whereas, for the 26 genomic fragments from the winter genotype Joelle the ratio was 1:4 for reads with the one base deletion (5 reads) vs. those containing the T (21 reads)~~1:4 for reads containing the T (21 reads) vs. those with the one base deletion (5 reads)~~; see positions 158-159 in Supplementary Figure 4. However, a Chi square test could not rule out a 1:5 ratio ($p=0.72$ with one degree of freedom)."
Figure 7 was also added to the manuscript to help clarify abundance of chromosome 20 *FLC* fragments obtained from RNAseq data that contain this specific mutation.

Discussion:

My main criticism of this manuscript is that new results are presented in the Discussion section, i.e. results are discussed in the Discussion section that were not previously presented in the text of the Results section. I mean the results with regards to transcript abundance of *GA3OX1*, *RGL1*, *RGL2*, *GID1A*, *GID1B*, *FT*, *TSF*, *TFL1*, VIN3, and *JMJD5*. See below

The Result section focuses on *FLC* and *MAF2*, but the results for the other genes included in Table 2 should also be included. It almost seems as if part of the Result section is missing. The data for

RGL1, *RGL2*, *GID1A* and *GID1B* have been removed from Table 2 and the Discussion; whereas, most text related to *GA3OX1*, *FT*, *TSF*, *TFL1*, *VIN3* and *JMJD5* was deleted from the Discussion and moved to appropriate sections of the Results.

On the other hand, *AGL24* is mentioned in the Introduction as a floral integrator, but not later in Results and Discussion (other than in Table 2). The following original sentences were moved from the Discussion to the Results section: "In arabidopsis, SOC1 also helps to regulate *AGL24* in response to GA (Lee and Lee 2010). Thus, the increased pre-vernalization abundance of *AGL24*, *SOC1* and a transcript involved in GA biosynthesis, *GA3OX1* (Table 2), in the summer annual genotype CO46 compared to the winter annual genotype Joelle would be consistent with the phenotypic responses (Fig. 1) observed in this study."

While *SVP* is discussed, the results of this study with regard to its transcript abundance are not included in the discussion. The results section was revised to read "At low temperatures, spliced variants of *MAF1* and *MAF2* produce proteins capable of interacting with the floral repressor SVP via direct binding to the vCArG III motif in the *FT* promoter (Lee et al. 2007). Although transcript abundance varied only slightly for *SVP* ~~A comparison of the~~ in ~~winter biotype~~ Joelle and ~~summer biotype~~ CO46 (Table 2), ~~indicates that~~ all three *MAF2* orthologues (Csa02g073630, Csa11g102220, Csa18g038750) did have increased transcript abundance (albeit below the 5 FPKM threshold) in Joelle relative to CO46 (Fig. 3C)."

The second paragraph of the Discussion section might benefit from a sub-heading, such as "Transcriptome profiles pre-and post vernalization" or something similar. Second paragraph of Discussion deleted or moved to Results section.

"Because a high degree of synteny and functionality has been …" Suggestions: " … and **similar** functionality…" Change made as suggested

"…transcript abundance of some classic floral regulators, such as FT, were minimal…". Please change to " .. **was** minimal…" (because it refers to transcript abundance). Done

Avoid the repeated referencing to Figures and Tables in the Discussion section. Revised as appropriate

Throughout the text, check for italicizing the gene IDs. Ok

Characterization of the three syntenic FLC loci:

"…, the increased abundance of FLC transcripts linked to Csa20g015400 in the winter genotype Joelle compared to summer genotypes CO46 and DH55 …". I don't think that it is valid to directly compare Joelle and CO46 to DH55, because the data for DH55 come from the transcriptome atlas and not from your own study where all plants were grown under the same conditions. Nevertheless, the data for DH55 can be used to support your hypothesis. The values for Csa8 are for example quite different in CO46 and DH55 (factor 2, almost 40 FPKM vs. under 20 for "young leaf"), which also suggests that a direct comparison should not be made. Noted and we revised the results to clarify the difference between Csa8 *FLC* abundance in leaf tissue of the two summer biotypes. We agree, as pointed out by the reviewer, that these are obviously different studies and should be viewed as such. Regardless, the reference transcriptome provides additional insights for building hypotheses; particularly considering that the DH55 reference genome was used to map transcriptome and whole genome reads from CO46

and Joelle in this study, as indicate in the Methods and Materials section.

" … consistent with the observed abundance of FLC alleles within our re-sequenced transcripts, which had a 1:5 and 5:1 ratio for T:-, respectively, in CO46 and Joelle…". This needs to be changed to **1:2 and 4:1**. This has been changed. Please see previous clarification above.

" … of the summer flowering biotype CO46 compared to winter biotype Joelle …" please change to "compared to **that of** winter biotype Joelle…" Done

"…chromosome 8 where a glutamine residue was absent at position 119 and a serine for arginine substitution occurred at position 132 in CO46 (Fig. 6)" This is also the case for DH55. Yes, see Figures 4 and 6, and Supplementary Figure 2 for clarification. This sentence was also revised to read "Another interesting divergence in predicted amino acid sequences between FLCs of the summer flowering biotypes CO46 and DH55 compared to that of winter biotype Joelle was observed for chromosome 8 where a glutamine residue was absent at position 119, and a serine for arginine substitution occurred at position 132 (Fig. 6)."
Did you check whether these changes have an effect on the functionality of the protein? The functional involvement of chromosome 8 *FLC* in the flowering process of camelina is being undertaken by another group.

Reviewer #2:

The statement "The vernalization-induced silencing of FLC occurs through the autonomous pathway (Berry and Dean 2015; Marquardt et al. 2014; McClung et al. 2016)" is just plain wrong. Indeed, one can have mutants in autonomous-pathway genes and the vernalization system works just fine! Moreover, to cite a McClung et al. 2016 review on 'The importance of ambient temperature to growth and the induction of flowering" for this is more than a bit odd. The authors might wish to consult some straightforward reviews such as the following:
Amasino RM, Michaels SD. 2010. The timing of flowering. Plant Physiol. 154:516-20.
Amasino R. 2010. Seasonal and developmental timing of flowering. Plant J. 61:1001-13.
Changed to read as follows: "The vernalization-induced silencing of *FLC* ~~occurs through the autonomous pathway~~ (Amasino 2010; Amasino and Michaels 2010; Berry and Dean 2015; Marquardt et al. 2014; ~~McClung et al. 2016), which~~ is believed to involve switching *FLC* activating histone marks (H3K4me3/H3K36me3/H2Bub1) to *FLC* de-activating histone marks (trimethylation of histone H3 at lysine 27; H3K27me3) by a protein complex that includes polycomb repressive complex 2 (PRC2) and the plant homeodomain (PHD) family (Berry and Dean 2015)."

In the Discussion the sections on 'Other factors that could play a role in the flowering habits of camelina' and 'Other factors that could play a role in the flowering habits of camelina' are tangential to the work and not necessary and read like a review article. Although some of the Discussion in these sections have been deleted, we believe that some of this Discussion is appropriate; not all readers will be as knowledgeable about the subject matter as the reviewers.

Along with the model plant arabidopsis (Arabidopsis Thaliana L.)
TO
Along with the model plant Arabidopsis (Arabidopsis thaliana L.) Done
and Cap 'A' for Arabidopsis throughout the paper is preferred but I do not think there is a definitive rule

on this. We believe "arabidopsis" is appropriate but have no issues with the editorial staff changing if necessary.

--- Editor comments

I have a couple of further suggestions on the manuscript

• In the methods section it is reported that the seeds were provided by Dr. Gesch who is one of the authors. Please indicate which was the initial source of the seeds as suggested by reviewer #1. See comments to reviewer #1 above.

• I found the description of the in the FLC one base deletion in the Abstract a bit confusing. It has to be clear that the manuscript describes the identification of a FLC allele that has a one base pair deletion that leads to a frameshift. The "resequencing of the genome" did not lead to identification of differences in the frequency of that allele. If I understood correctly that was done by PCR amplification and sequencing of the FLC gene. To help clarify, we have revised the Abstract to read "The nature of the vegetative to reproductive transition in the shoot apical meristem of *Camelina sativa* summer annual cultivar CO46 and winter annual cultivar Joelle was confirmed by treating seedlings with or without 8 weeks of vernalization. True to their life cycle classification, Joelle required a vernalization treatment to induce bolting and flowering, whereas CO46 did not. In this study, whole genome sequence, RNAseq, and resequencing of PCR amplified transcripts for a key floral repressor were used to better understand factors involved in the flowering habit of summer- and winter-biotypes at the molecular level. Analysis of transcriptome data indicated that abundance for one of the three genes encoding the floral repressor *FLOWERING LOCUS C* (*FLC*; Csa20g258400) was 16-fold greater in Joelle compared to CO46 prior to vernalization. Abundance of this transcript decreased only slightly in CO46 post-vernalization, compared to a substantial decrease in Joelle. The results observed in the winter annual biotype Joelle are consistent with repression of *FLC* by vernalization. Further characterization of *FLC* at both the genome and transcriptome levels identified a one base deletion in the 5th exon coding for a keratin-binding domain in chromosome 20 of CO46 and Joelle. The one base deletion detected in chromosome 20 *FLC* is predicted to result in a frame shift that would produce a non-functional protein. Analysis of whole genome sequence indicated that the one base deletion in chromosome 20 *FLC* occurred at a greater ratio in the summer biotype CO46 (2:1) compared to the winter biotype Joelle (1:4); similar trends were also observed for RNAseq and cDNA transcripts mapping to chromosome 20 *FLC* of CO46 and Joelle."

• It would be also useful to the reader to explain that Camelina has a hexaploid genome structure due to a recent genome triplication although it shows diploid inheritance. The triplication explains the presence of three copies of the FLC gene. The following sentence of Introduction was revised to help clarify this point "Camelina is a hexaploid (2n=40) with an estimated genome size of ~782 Mb that is believed to have originated from an allotetraploid sub-genome with seven chromosomes each and a diploid sub-genome with six chromosomes (Berti et al. 2016; Kagale et al., 2014)."
