# Supplementary material for: Expression of FLOWERING LOCUS C and a frameshift mutation of this gene on chromosome 20 differentiate a summer and winter annual biotype of Camelina sativa
Source: Plant Direct. 2018 Jul 9;2(7):e00060. doi: 10.1002/pld3.60 (PMC6508819; doi:10.1002/pld3.60)

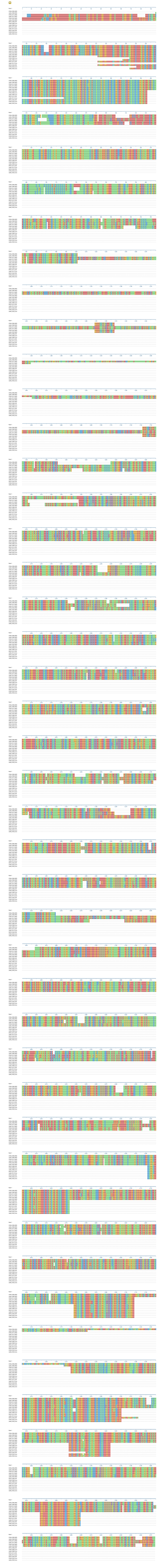
Ruler 1
CO46_Ch08_WGS
Joelle_Ch08_WGS
CO46_Ch13_WGS
Joelle_Ch13_WGS
CO46_Ch20_WGS
Joelle_Ch20_WGS
DH55_Ch08_CDS
CO46_Ch08_Cons
Joelle_Ch08_Cons
DH55_Ch13_CDS
Joelle_Ch13_Cons
DH55_Ch20_CDS
CO46_Ch20_Cons
Joelle_Ch20_Cons

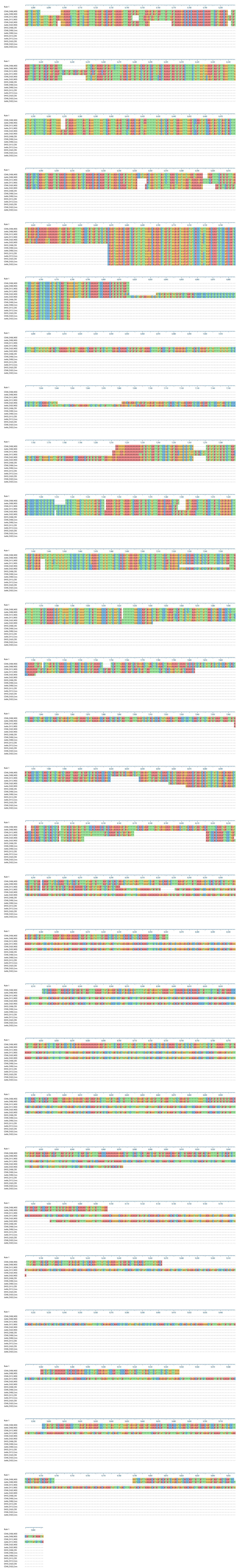

Supplement: Supplementary file 2 [file PLD3-2-e00060-s002.pdf]
